# Monitoring COVID‐19 vaccine effectiveness against COVID‐19 hospitalisation and death using electronic health registries in ≥65 years old population in six European countries, October 2021 to November 2022

**DOI:** 10.1111/irv.13195

**Published:** 2023-11-28

**Authors:** Irina Kislaya, Alexis Sentís, Jostein Starrfelt, Baltazar Nunes, Iván Martínez‐Baz, Katrine Finderup Nielsen, Ala'a AlKerwi, Toon Braeye, Mario Fontán‐Vela, Sabrina Bacci, Hinta Meijerink, Jesús Castilla, Hanne‐Dorthe Emborg, Christian Holm Hansen, Susanne Schmitz, Izaak Van Evercooren, Marta Valenciano, Anthony Nardone, Nathalie Nicolay, Susana Monge, VEBIS‐Lot4 working group, Bolette Søborg, Joris A. F. van Loenhout, Ausenda Machado, Palle Valentiner‐Branth, Carlos Dias, Pierre Hubin, Itziar Casado, Aitziber Echeverría, Cristina Burgui, Amparo Larrauri, Esther Kissling, Marine Maurel, Liliana Antunes, Matylde Diouf

**Affiliations:** ^1^ Department of Epidemiology Instituto Nacional de Saúde Doutor Ricardo Jorge (INSA) Lisbon Portugal; ^2^ Epidemiology Department Epiconcept Paris France; ^3^ Norwegian Institute of Public Health (NIPH) Oslo Norway; ^4^ Instituto de Salud Pública de Navarra ‐ IdiSNA Pamplona Spain; ^5^ CIBER on Epidemiology and Public Health (CIBERESP) Madrid Spain; ^6^ Department of Infectious Disease Epidemiology & Prevention Statens Serum Institut (SSI) Copenhagen Denmark; ^7^ Ministry of Health, Directorate of Health, Service Epidemiology and Statistics Luxembourg Luxembourg; ^8^ Epidemiology of infectious diseases, Sciensano Brussels Belgium; ^9^ Instituto de Salud Carlos III (ISCIII), Madrid Spain; ^10^ Public Health and Epidemiology research group School of Medicine and Health Sciences, Universidad de Alcalá, Alcalá de Henares Madrid Spain; ^11^ Vaccine Preventable Diseases and Immunisation European Centre for Disease Prevention and Control (ECDC) Solna Sweden; ^12^ CIBER on Infectious Diseases (CIBERINFEC) Madrid Spain

**Keywords:** cohort design, COVID‐19, COVID‐19‐related death, hospitalization, SARS‐CoV‐2, vaccine effectiveness

## Abstract

**Background:**

Within the ECDC‐VEBIS project, we prospectively monitored vaccine effectiveness (VE) against COVID‐19 hospitalisation and COVID‐19‐related death using electronic health registries (EHR), between October 2021 and November 2022, in community‐dwelling residents aged 65–79 and ≥80 years in six European countries.

**Methods:**

EHR linkage was used to construct population cohorts in Belgium, Denmark, Luxembourg, Navarre (Spain), Norway and Portugal. Using a common protocol, for each outcome, VE was estimated monthly over 8‐week follow‐up periods, allowing 1 month‐lag for data consolidation. Cox proportional‐hazards models were used to estimate adjusted hazard ratios (aHR) and VE = (1 − aHR) × 100%. Site‐specific estimates were pooled using random‐effects meta‐analysis.

**Results:**

For ≥80 years, considering unvaccinated as the reference, VE against COVID‐19 hospitalisation decreased from 66.9% (95% CI: 60.1; 72.6) to 36.1% (95% CI: −27.3; 67.9) for the primary vaccination and from 95.6% (95% CI: 88.0; 98.4) to 67.7% (95% CI: 45.9; 80.8) for the first booster. Similar trends were observed for 65–79 years. The second booster VE against hospitalisation ranged between 82.0% (95% CI: 75.9; 87.0) and 83.9% (95% CI: 77.7; 88.4) for the ≥80 years and between 39.3% (95% CI: −3.9; 64.5) and 80.6% (95% CI: 67.2; 88.5) for 65–79 years. The first booster VE against COVID‐19‐related death declined over time for both age groups, while the second booster VE against death remained above 80% for the ≥80 years.

**Conclusions:**

Successive vaccine boosters played a relevant role in maintaining protection against COVID‐19 hospitalisation and death, in the context of decreasing VE over time. Multicountry data from EHR facilitate robust near‐real‐time VE monitoring in the EU/EEA and support public health decision‐making.

## 1 INTRODUCTION

In December 2020, almost 1 year into the coronavirus disease 2019 (COVID‐19) pandemic, the first vaccines against severe acute respiratory syndrome coronavirus 2 (SARS‐CoV‐2), BNT162b2 (Comirnaty®), mRNA‐1273 (Spikevax®), ChAdOx1 (Vaxzevria®) and Ad26.COV2‐S (JCovden) received early conditional marketing authorisation from the European Medicines Agency (EMA).^1^ These vaccines were developed against the original strain of SARS‐CoV‐2, used in the initial phase of the vaccination campaign and showed high vaccine effectiveness (VE) that, however, waned over time.^2^, ^3^ The mRNA vaccines (Comirnaty® and Spikevax®) were then deployed as the first booster administered during the Autumn of 2021 and as the second booster in the Spring of 2022 in some countries, prioritising individuals of older age and those with medical underlying conditions, and further extending to other age groups. Four adapted mRNA vaccines targeting Omicron subvariants (Comirnaty® bivalent Original/Omicron BA.1, Comirnaty® bivalent Original/Omicron BA.4‐5, Spikevax® bivalent Original/Omicron BA.1, Spikevax® bivalent Original/Omicron BA.4‐5) were further authorised and administered from September 2022 onwards for second and third booster vaccination in the European Union/European Economic Area (EU/EEA).^1^, ^4^, ^5^

Following the rollout of mass COVID‐19 vaccination programmes, real‐world VE monitoring started to estimate the level and duration of protection, the VE in specific populations not covered by clinical trials and VE against new emerging genetic variants of SARS‐CoV‐2.^2^, ^3^, ^6^, ^7^, ^8^ Rapid availability of this information has been of great value in guiding public health decision‐makers to adapt vaccination programmes according to public health needs. The use of population‐based Electronic‐Health Registries (EHR) has the advantage of large sample sizes and being readily available, allowing prospective timely monitoring of VE and with relatively few extra resources. Because of this, EHR has become a core data source for COVID‐19 VE studies in many countries.^2^, ^3^, ^7^, ^9^, ^10^, ^11^, ^12^, ^13^

By the end of 2021, the European Centre for Disease Prevention and Control (ECDC) established the Vaccine Effectiveness, Burden and Impact Studies of COVID‐19 and Influenza (VEBIS) project to monitor the effectiveness of vaccines in real‐world conditions and to inform public health actions and adaptation of vaccination programmes in the EU/EEA countries. One component of VEBIS is based on estimating VE using routinely collected vaccination and outcome data from EHR.^14^ This project is leveraging established vaccination registries and health record databases using the combination of mandatory reporting of the vaccination status into these registries and a unique personal identification number or a unique social security number across health databases as the key for individual level data linkage, allowing to perform VE studies.^2^, ^10^, ^11^, ^15^, ^16^

The overall aim of this component of the VEBIS project is to expand the use of electronic health registries across EU/EEA and to establish robust statistical methods to monitor COVID‐19 VE over time as well as to improve the timeliness of reporting of VE estimates across EU/EEA. With this aim, we carried out a multi‐country study based on EHR to prospectively provide monthly VE estimates of the complete primary vaccination series, the first, the second, and the third booster dose against COVID‐19 hospitalisation and COVID‐19‐related death, in community‐dwelling resident population aged 65 years and over.

## 2 METHODS

### 2.1 Study design and setting

Using a common protocol,^14^ Belgium, Denmark, Luxembourg, Navarre (Spain), Norway, and Portugal constructed population cohorts based on data collected routinely in EHR. Countries were recruited based on their assessed capability to join the study and a formal outreach performed by the ECDC. We used individual deterministic linkage to cross‐match administrative population and statistical office databases with registers for COVID‐19 vaccination, SARS‐CoV‐2 testing, hospitalisations, deaths and clinical data. Belgium was unable to include unvaccinated individuals but followed the same protocol to compare individuals with different vaccination statuses to provide relative VE (rVE) estimates. A description of the EHR used by each study site to monitor COVID‐19 VE is provided in the Supporting Information (Appendix S1).

We estimated COVID‐19 VE with monthly frequency. For each month, the observation period covered 8 weeks to allow sufficient events to provide precise estimates and to be sensitive to changes in VE over time. Overall, the observation period was between October 2021 and November 2022. Between October 2021 and March 2022, we piloted a common protocol for an outcome of COVID‐19 hospitalisation in four study sites: Denmark, Navarre (Spain), Norway, and Portugal.^17^ From March–April 2022 onwards, after the validation of the pilot study with the COVID‐19 hospitalisation outcome, the outcome of COVID‐19‐related death was incorporated. From April–May 2022 onwards, we added rVE estimates and, from July–August 2022 onwards, we added two study sites (Belgium and Luxembourg).

### 2.2 Selection criteria and definitions

We included individuals aged between 65 and 110 years (inclusive). We excluded residents in long‐term‐care facilities (using the last available information) and early vaccinees (defined as either being vaccinated before recommended for their age group or the first 5% vaccinated of each 5‐year age band) as these were considered especially vulnerable groups with different probabilities of vaccination and developing severe COVID‐19. In addition, we excluded those with inconsistent data on vaccination (two‐dose primary vaccination with <19 days apart, or those vaccinated with a booster <90 days after the last primary or booster dose, or a combination of brands other than recommended in the EU/EAA). Individuals with a previously recorded positive SARS‐CoV‐2 test (previous infection) were excluded until April 2022, but not thereafter. During the Omicron wave, there was a very high incidence, which was largely under‐reported due to the discontinuation of systematic testing in most countries and the widespread use of self‐tests. In order to reduce misclassification and increase the generalisability of results, the restriction to individuals without documented infection was removed for estimates from April–May 2022 onwards.

Outcomes of interest were (1) hospitalisation due to COVID‐19, defined as admission to a hospital with a SARS‐CoV‐2 infection laboratory‐confirmed from 14 days before to 1 day after admission, in which admission criteria are compatible with a severe acute respiratory infection,^18^, ^19^ or in which COVID‐19 is the main diagnosis in the discharge record, and (2) COVID‐19‐related death, defined as death for which COVID‐19 is recorded as the cause of death (even with no positive SARS‐CoV‐2 test recorded in the EHR) or, if the cause of death is not available, laboratory‐confirmed SARS‐CoV‐2 infection with death in the 30 days after the positive test.

We assumed as the date of the outcome the minimum between the date of the positive test result (i.e., the date of the first SARS‐CoV‐2 positive test of the infection episode that resulted in hospital admission or death) and the date of hospital admission or death (respectively for each outcome).

The vaccination status was defined as a time‐varying variable within each observation period and classified as follows: (1) unvaccinated (no record of COVID‐19 vaccine administration); (2) complete primary vaccination, if received one dose of Jcovden® or two doses of any combination of Comirnaty®, Spikevax® or Vaxzevria®; (3) vaccination with the first booster, if received an additional dose of Comirnaty®, Spikevax® (monovalent or bivalent) at least 90 days after the complete primary vaccination; (4) vaccination with the second booster, if received a first booster and an additional dose of Comirnaty®, Spikevax® (monovalent or bivalent) at least 90 days later; (5) vaccination with the third booster, defined in the same way as the second booster.

### 2.3 Analytical methods for site‐specific vaccine effectiveness estimates

We used a survival analysis framework with calendar time as the underlying time scale, assigning time zero to the first day of each observation period. We excluded person‐time at‐risk from the date of receipt of any vaccine dose until 13 days after. Follow‐up started at the beginning of the observation period and ended at the earliest occurrence of any of the following events: (1) outcome of interest, (2) death of any cause, (3) discontinuation in the administrative database (e.g., emigration), or (4) administrative censoring (8 weeks after time zero).

We estimated confounder adjusted hazard ratios (aHR) of each outcome and 95% confidence intervals (CI) with Cox proportional hazards models. The adjustment variables included sex, age group (in 5‐year age bands), previous SARS‐CoV‐2 infection, comorbidities (with the exception of Luxembourg) and other variables relevant at each study site (Appendix S2). We computed VE as (1 − aHR) × 100%, using the unvaccinated as the reference group in the primary analysis. To estimate the rVE of booster doses, we considered those with complete primary vaccination ≥169 days ago as well as those with first booster ≥90 days ago as a reference, since those groups were eligible for the booster uptake. All estimates were stratified by age group (65–79 or ≥80 years old).

For data protection reasons, sites reported aHR estimates only when at least five events per vaccination status category were observed. All sites fulfilled ethical and data protection requirements according to their national legislation (Appendix S3).

### 2.4 Analytical methods for pooled vaccine effectiveness estimates

We used a random‐effects meta‐analysis (Paule‐Mandel method)^20^ to pool site‐specific aHRs, accounting for within and between sites variability in the estimates. The number of sites contributing to the pooled analysis for the different vaccination statuses at each follow‐up period varied due to differences in the national COVID‐19 vaccination campaign rollout (Appendix S4). Only sites with general recommendations of respective doses in 65–79 years and ≥80‐year‐old were included in each 8‐week observation period. Pooled VE estimates obtained with fewer than 15 events were not reported.

## 3 RESULTS

### 3.1 Characteristics of the study population

**FIGURE 1:**
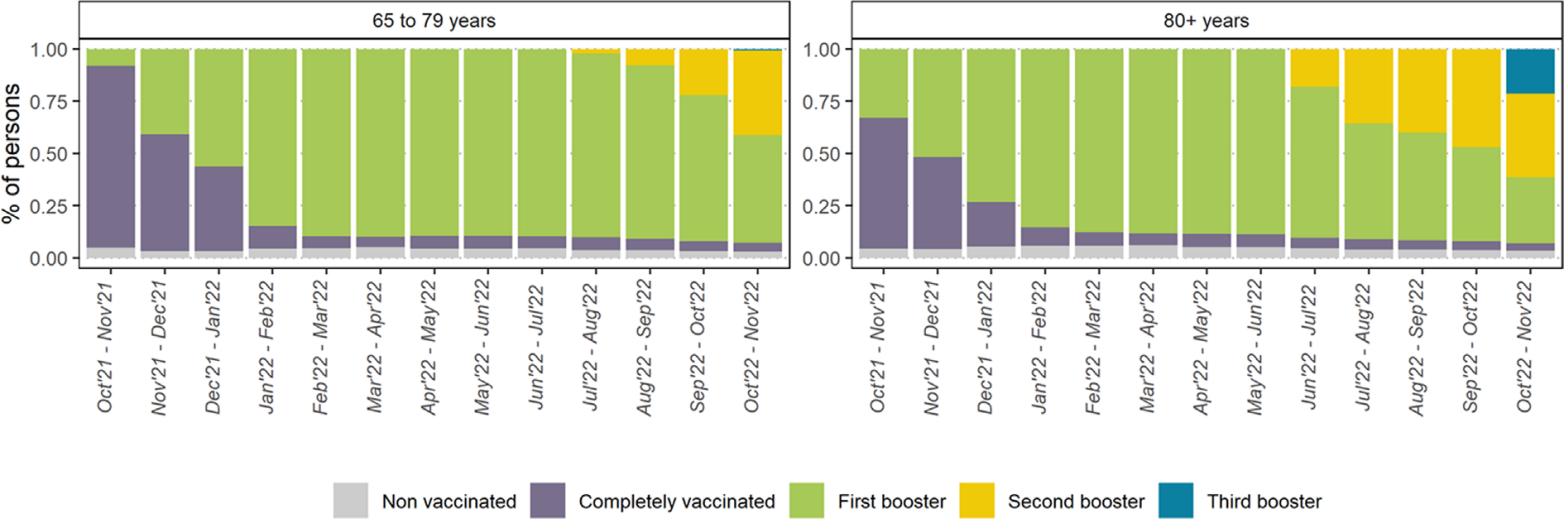
Proportion of the study population by vaccination status and observation period, from October 2021 to November 2022.

Pooling together the data from the four participating study‐sites up to June–July 2022 and from the six participating study‐sites thereon, the distribution of study participants by vaccination status in each observation period is shown in Figure 1. Throughout the study, the proportion of unvaccinated individuals remained low, at 3–5% of 65–79 years and 1–2% of ≥80 years. The proportion of vaccinated with the first booster increased progressively in both age groups until the respective second booster recommendation and declined afterwards. In the last observation period (October–November 2022), 25% of ≥80 years had been vaccinated with the third booster.

The number of observed COVID‐19 hospitalisations ranged between 1045 in the first observation period (October–November 2021) and 475 in the last observation period (October–November 2022) among 65–79 years, and between 820 and 687 among ≥80 years (Appendix S5, Table S1). The number of COVID‐19‐related deaths registered among 65–79 years ranged between 608 in March–April 2022 and 252 in October–November 2022 and between 1605 and 505 among the ≥80 years (Appendix S5, Table S2).

### 3.2 Vaccine effectiveness against COVID‐19 hospitalisation (vs. unvaccinated)

**FIGURE 2:**
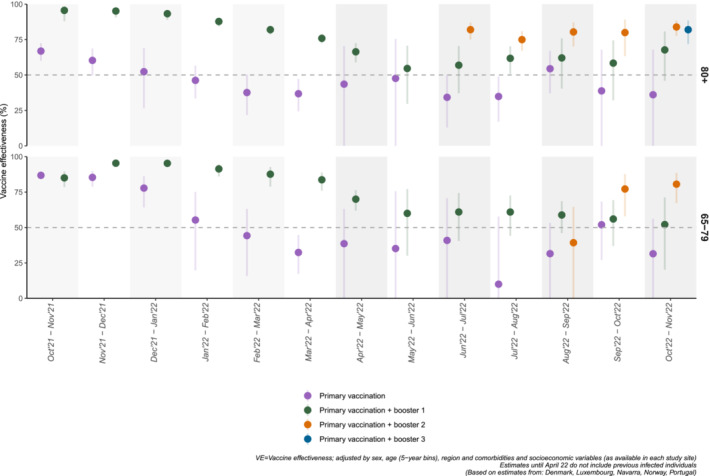
Pooled primary vaccination and booster dose(s) vaccine effectiveness against COVID‐19 hospitalization by age group, in overlapping 8‐week wide observation intervals from October 2021 to November 2022, in six EU/EEA countries. Random effects meta‐analysis.

For the 65–79 years, VE of complete primary vaccination against COVID‐19 hospitalisation declined from 86.8% (95% CI: 84.5; 88.8) in October–November 2021 to 31.5% (95% CI: −7.1; 56.2) in October–November 2022. For the ≥80 years, a similar trend was observed VE of complete primary vaccination against COVID‐19 hospitalisation decreased from 66.9% (95% CI: 60.1; 72.6) to 36.1% (95% CI: −27.3; 67.9) (Table 1, Figure 2).

**TABLE 1:** Estimated vaccine effectiveness (VE) for complete primary vaccination (vs. unvaccinated) against COVID‐19 hospitalisation by age group, in overlapping 8‐week wide observation intervals from October 2021 to November 2022 in five EU/EEA countries. Random effects meta‐analysis.

Age group	VE (95% CI) vs. unvaccinated												
	October 1 to November 25, 2021^a^	November 1 to December 26, 2021^a^	December 1, 2021, to January 25, 2022^a^	January 1 to February 25, 2022^a^	February 1 to March 28, 2022^a^	March 1 to April 25, 2022^a^	April 1 to May 26, 2022^a^	May 1 to June 25, 2022^a^	June 1 to July 26, 2022^a^	July 1 to August 25, 2022	August 1 to September 25, 2022	September 1 to October 26, 2022	October 1, 2022, to November 25, 2022
65–79 year‐olds	86.8% (84.5; 88.8)	85.4% (78.8; 89.9)	77.8% (64.2; 86.3)	55.3% (19.7; 75.1)	44.3% (15.7; 63.1)	32.4% (17.3; 44.7) ^b^	38.6% (−2.3; 63.2)	35.2% (−72.4; 75.6)^b^ ^,^ ^c^	40.9% (−19.0; 70.7)	10.0% (−91.7; 57.8)^d^	31.6% (−0.2; 53.2)^b^ ^,^ ^d^	52.0% (27.1; 68.3)^b^ ^,^ ^d^	31.5% (−7.1; 56.2)^b^ ^,^ ^d^ ^,^ ^e^
≥80‐year‐olds	66.9% (60.1; 72.6)	60.3% (49.7; 68.7)	52.4% (26.5; 69.1)	46.2% (33.4; 56.6)	37.6% (21.7; 50.3)^b^	36.8% (24.4; 47.2)^b^	43.5% (−8.0; 70.5)^b^	47.6% (−11.6; 75.4)^b^ ^,^ ^c^	34.3% (12.9; 50.3)	34.9% (17.0; 48.9)^d^	54.5% (37.1; 67.0)^b^ ^,^ ^d^	38.8% (−16.5; 67.8)^b^ ^,^ ^d^	36.1% (−27.3; 67.9)^b^ ^,^ ^d^

Abbreviations: CI, confidence interval; VE, vaccine effectiveness.

^a^ Unless otherwise indicated, results up to June 1 to July 26, 2022, are limited to four sites: Denmark, Navarre (Spain), Norway and Portugal.

^b^ Navarre (Spain) did not reach five events and did not contribute to the estimate.

^c^ Denmark did not reach five events and did not contribute to the estimate.

^d^ Luxembourg did not reach five events and did not contribute to the estimate.

^e^ Norway did not reach five events and did not contribute to the estimate.

For the 65–79 years, the first booster VE declined progressively from 95.4% (95% CI: 92.9; 97.0) in November–December 2021 to 52.1% (95% CI: 20.2; 71.2) in October–November 2022. VE of the first booster for the ≥80 years peaked at 95.6% (95% CI: 88.0; 98.4) in October–November 2021 declining to 54.6% (95% CI: 29.6; 70.7) by May 2022 with little variation afterwards (Table 2).

**TABLE 2:** Vaccine effectiveness (VE) and relative vaccine effectiveness (rVE) for the first booster dose against COVID‐19 hospitalisation by age group, in overlapping 8‐week wide observation intervals from October 2021 to November 2022 in six EU/EEA countries. Random effects meta‐analysis.

Age group	October 1 to November 25, 2021^a^	November 1 to December 26, 2021^a^	December 1, 2021, to January 25, 2022^a^	January 1 to February 25, 2022^a^	February 1 to March 28, 2022^a^	March 1 to April 25, 2022^a^	April 1 to May 26, 2022^a^	May 1 to June 25, 2022^a^	June 1 to July 26, 2022^a^	July 1 to August 25, 2022	August 1 to September 25, 2022	September 1 to October 26, 2022	October 1, 2022, to November 25, 2022
	**VE (95% CI) vs. unvaccinated**												
65–79 year‐olds	85.0% (78.6; 89.5)^b^ ^,^ ^c^	95.4% (92.9; 97.0)	95.3% (93.8; 96.5)	91.4% (86.0; 94.7)	87.6% (78.8; 92.8)	83.7% (76.0; 88.9)	70.0% (61.9; 76.4)	60.0% (30.1; 77.1)	61.0% (40.5; 74.4)	60.9% (44.2; 72.7)	58.8% (46.0; 68.6)	56.0% (36.9; 69.4)	52.1% (20.2; 71.2)
≥80‐year‐olds	95.6% (88.0; 98.4)	95.2% (90.6; 97.5)	93.3% (88.9; 95.9)	87.8% (84.4; 90.4)	82.0% (78.6; 84.9)	75.9% (72.4; 78.9)	66.4% (59.0; 72.5)	54.6% (29.6; 70.7)	56.9% 37.1; 70.5)	61.8% (50.8; 70.3)	62.1% (40.4; 75.9)^d^	58.4% (32.1; 74.5)	67.7% (45.9; 80.8)
	**rVE (95% CI) vs. complete primary vaccination ≥169 days ago**												
65‐ to 79‐year‐olds	N/A	N/A	N/A	N/A	N/A	N/A	64.2% (42.2; 77.8)	47.1% (33.6; 57.8)	34.6% 19.2; 47.0)	47.5% (30.6; 60.3)	43.2% (20.6; 59.3)	18.6% (−8.1; 38.7)^b^	30.4% (9.8; 46.3)^b^
≥80‐year‐olds	N/A	N/A	N/A	N/A	N/A	N/A	52.2% (24.7; 69.6)	42.2% (32.6; 50.5)	44.1% (29.4; 55.7)	37.5% (16.0; 53.5)	28.9% (11.8; 42.7)^d^	32.2% (14.9; 46)^b^	45.5% (31.4; 56.7)

Abbreviations: CI, confidence interval; N/A, not applicable: In the first 6 months of the study rVE was not estimated; VE, vaccine effectiveness.

^a^ Unless otherwise indicated, results up to June 1 to July 26, 2022, are limited to four sites: Denmark, Navarre (Spain), Norway and Portugal.

^b^ Navarre (Spain) did not reach five events and did not contribute to the estimate.

^c^ Portugal did not reach five events and did not contribute to the estimate.

^d^ Luxembourg did not reach five events and did not contribute to the estimate.

The second booster VE for the 65–79 years was 39.3% (95% CI: −3.9; 64.5) in August–September 2022 but increased to 80.6% (95% CI: 67.2; 88.5) in October–November 2022. For the ≥80 years, VE started at 82.0% (95% CI: 75.9; 87.0) in June–July 2022 and was 83.9% (95% CI: 77.7; 88.4) in October–November 2022 (Table 3).

**TABLE 3:** Estimates of vaccine effectiveness (VE) and relative vaccine effectiveness (rVE) for the second booster dose against COVID‐19 hospitalisation by age group, in overlapping 8‐week wide observation intervals from May 2021 (earliest month with available estimates for the 2nd booster) to November 2022, in six EU/EEA countries. Random effects meta‐analysis.

Age group	June 1 to July 26, 2022^a^	July 1 to August 25, 2022	August 1 to September 25, 2022	September 1 to October 26, 2022	October 1, 2022, to November 25, 2022	
	**VE (95% CI) vs. unvaccinated**				
65‐ to 79‐year‐olds	N/A	N/A	39.3% (−3.9; 64.5)^b^ ^,^ ^c^ ^,^ ^d^ ^,^ ^e^	77.2% (57.9; 87.7)^b^ ^,^ ^c^ ^,^ ^e^	80.6% (67.2; 88.5)^b^ ^,^ ^c^
≥80‐year‐olds	82.0% (75.0; 87.0)^b^ ^,^ ^c^ ^,^ ^d^ ^,^ ^f^	75.0% (67.1; 81.0)^b^ ^,^ ^c^ ^,^ ^d^	80.4% (70.1; 87.1)^b^ ^,^ ^c^ ^,^ ^d^	80.0% (63.3; 89.0)^b^ ^,^ ^c^	83.9% (77.7; 88.4)^b^ ^,^ ^c^
	**rVE (95% CI) vs. complete primary vaccination ≥169 days ago**				
65‐ to 79‐year‐olds	N/A	N/A	22.7% (−29.6; 53.9)^b^ ^,^ ^c^ ^,^ ^d^ ^,^ ^e^	51.2% (9.9; 73.6)^b^	73.4% (62.2; 81.3)^b^ ^,^ ^c^
≥80‐year‐olds	71.0% (61.4; 78.2)^b^ ^,^ ^c^ ^,^ ^d^ ^,^ ^f^	57.8% (48.2; 65.6)^b^ ^,^ ^c^ ^,^ ^d^	57.2% (43.1; 67.9)^b^ ^,^ ^c^ ^,^ ^d^	50.7% (35.8; 62.2)^b^ ^,^ ^c^	68.4% (54.5; 78.1)^b^
	**rVE (95% CI) vs. the first booster≥90 days ago**				
65‐ to 79‐year‐olds	N/A	N/A	−39.9% (−89.3; 3.4)^b^ ^,^ ^c^ ^,^ ^d^ ^,^ ^e^	33.3% (9.5; 50.8)^b^ ^,^ ^c^ ^,^ ^e^	57.4% (34.6; 72.2)^b^ ^,^ ^c^
≥80‐year‐olds	54.0% (42.9; 62.9)^b^ ^,^ ^c^ ^,^ ^d^ ^,^ ^f^	41.6% (11.1; 61.6)^b^ ^,^ ^c^ ^,^ ^d^	42.2% (9.7; 63.0)^b^ ^,^ ^c^ ^,^ ^d^	34.2% (16.8; 47.9)^b^ ^,^ ^c^	47.0% (12.5; 67.9)^b^

Abbreviations: CI, confidence interval; N/A, not applicable before vaccine recommendation was issued; VE, vaccine effectiveness.

^a^ Unless otherwise indicated, results up to June 1 to July 26, 2022, are limited to four sites: Denmark, Navarre (Spain), Norway and Portugal.

^b^ Navarre (Spain) did not reach five events or this dose was still not recommended at this site and did not contribute to the estimate.

^c^ Denmark did not reach five events or this dose was still not recommended at this site and did not contribute to the estimate.

^d^ Luxembourg did not reach five events or this dose was still not recommended at this site and did not contribute to the estimate.

^e^ Portugal did not reach five events or this dose was still not recommended at this site and did not contribute to the estimate.

^f^ Norway did not reach five events or this dose was still not recommended at this site and did not contribute to the estimate.

The third booster VE estimate against COVID‐19 hospitalisation was 82.0% (95% CI: 71.8; 88.5) but was only available for ≥80 years in October–November 2022 in Portugal.

### 3.3 Relative vaccine effectiveness (rVE) against COVID‐19 hospitalisation

Relative to complete primary vaccination ≥169 days ago, the first booster rVE for the 65–79 years varied between 64.2% (95% CI: 42.2; 77.8) in April–May 2022 and 30.4% (95% CI: 9.8; 46.3) in October–November 2022. The corresponding estimates for the ≥80 years were 52.2% (95% CI: 24.7; 69.6) and 45.5% (95% CI: 31.4; 56.7), respectively. (Table 2; Appendix S6, Figure S1).

For the 65–79 years, relative to the first booster vaccination ≥90 days ago, the incremental protection conferred by the second booster varied, from −39.9% (95% CI: −89.3; 3.4) in August–September 2022 to 57.4% (95% CI: 34.6; 72.2) in October–November 2022. For the ≥80 years rVE of the second booster, introduced in late spring of 2022 ranged between 54.0% (95% CI: 42.9; 62.9) in June–July 2022 and 34.2% (95% CI: 16.8; 47.9) in September–October 2022., (Table 3; Appendix S6, Figure S2).

### 3.4 Vaccine effectiveness against COVID‐19‐related death (vs. unvaccinated)

**FIGURE 3:**
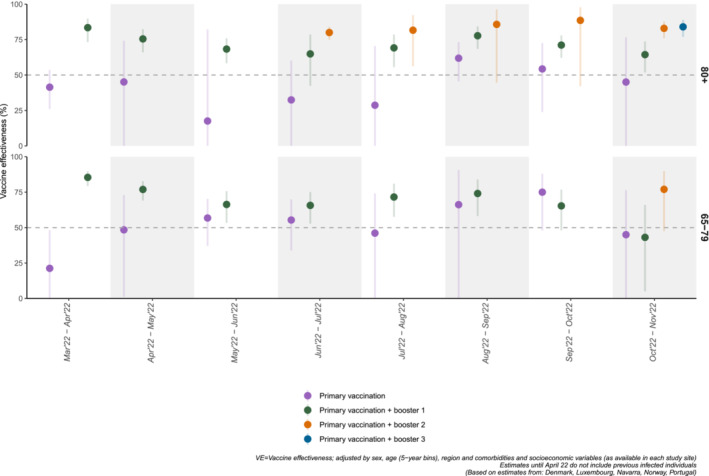
Pooled primary vaccination and booster dose(s) vaccine effectiveness against COVID‐19 death by age group, in overlapping 8‐week wide observation intervals from March 2022 to November 2022, in six EU/EEA countries. Random effects meta‐analysis.

For the 65–79 years, complete primary vaccination VE against COVID‐19‐related death varied between 21.3% (95% CI: −19.8; 48.2) in March–April 2022 and 45.0% (95% CI: −28.0; 76.4) in October–November 2022. The corresponding figures for ≥80 years were 41.4% (95% CI: 26.0; 53.6) and 45.1% (95% CI: −29.2; 76.6) (Figure 3; Appendix S7, Table S3).

We observed a decrease in the first booster VE estimates between March–April 2022 and October–November 2022 from 85.4% (95% CI: 79.3; 89.8) to 43.1% (95% CI: 4.9; 66.0) for the 65–79 years and from 83.5% (95% CI: 73.2; 89.8) to 64.4% (95% CI: 51.8; 73.7) for the ≥80 years (Figure 2; Appendix S7, Table S4).

For the 65–79 years, second booster VE was 77.0% (95% CI: 47.6; 89.9) in the last observation period (October–November 2022); for ≥80 years, it remained above 80% between June–July 2022 and October–November 2022, showing no evident trends (Table S5, Figure 2).

### 3.5 Relative vaccine effectiveness (rVE) against COVID‐19‐related death

rVE estimates showed that additional protection against COVID‐19‐related death achieved with the first booster decreased over time in both age groups (Appendix S7, Table S4, Figure S3), specifically from 66.0% (95% CI: 55.2; 74.3) in April–May 2022 to −8.5% (95% CI: −69.6; 30.6) in October–November 2022 for the 65–79 years and respectively from 61.3% (95% CI: 48.3; 71.0) to 34.6% (95% CI: 10.7; 52.1) for the ≥80 years.

For the 65–79 years, the first available estimate of second booster rVE (vs. first booster vaccination ≥90 days ago) was 74.0% (95% CI: 60.6; 82.8) in October–November 2022 (Appendix S7, Table S5, Figure S4). For the ≥80 years, the second booster rVE against COVID‐19‐related death varied between 58.0% (95% CI: 50.3; 64.5) in June–July 2022 and 65.0% (95% CI: 40.9; 79.3) in October–November 2022.

## 4 DISCUSSION

The prospective production of VE estimates using population‐based EHR with short time lag between data consolidation and data analysis is an added value to provide necessary evidence to adapt vaccine policies in the different target groups in a timely way.^1^, ^4^, ^5^ In this study, timely, rapid and robust estimates have been calculated using a common protocol applied to population registries for complete primary vaccination, first, second and third booster doses. The harmonization of the outcome and exposure definitions, and the application of common analytical methods enhanced comparability and allowed for joint estimates. These pooling methodological approaches are of high added value especially when the incidence of COVID‐19 decreases and fewer events are reported. Results are based on a multi‐country collaboration and estimates reflect on the performance of the vaccines in the population across several countries. In addition, the overall study period covered the predominance of the Delta SARS‐COV‐2 variant, the emergence of the Omicron and its subvariants, as well as the successive administration of first, second and third vaccine boosters,^4^, ^21^ which is another key strength of this analysis. Nonetheless, the production of real‐time VE estimates depends on access approvals to different EHR by the public health institutes. While such access has been relatively easily granted in exceptional circumstances during the pandemic, the sustainability of such process may prove difficult in the future.

Our results showed a decrease in complete primary vaccination VE against hospitalisation in both age groups (65–79 and ≥80 years) from 87%–67% in October–November 2021 to 32%–36% in October–November 2022. While the first booster initially restored immunity to similar levels to the ones observed at the beginning of the vaccination programme (≥95% by the end of 2021), its VE also decreased to approximately 50%–68% by May 2022, around 6–7 months after first booster vaccination campaign and after the emergence of Omicron and its subvariants. VE estimates against COVID‐19‐related deaths of the first booster, available since March–April 2022, showed a similar trend, although less pronounced among ≥80 years, compared to 65–79 years.

The significant decline in VE following the emergence of SARS‐CoV‐2 Omicron in December 2021 is in line with neutralisation studies indicating vaccine escape by Omicron.^22^, ^23^ It is also highly consistent with reports from the United States, Canada, South Africa and Europe^6^, ^7^, ^8^, ^24^ on lower VE against severe form of the disease during the Omicron subvariants predominance, in particular BA.2 and BA.4/BA.5. Rapid waning of first booster VE against hospitalisation during Omicron predominant period has also been reported in the literature (VE of 29%–58% 3–6 months after uptake).^6^, ^8^ This decline in VE motivated the recommendation for an additional booster dose in vulnerable population subgroups, but also the development of adapted vaccines to closely match circulating variants.

Other factors could also contribute to the observed decrease in VE. The Omicron BA.1 wave in early 2022 resulted in the highest SARS‐CoV‐2 incidence observed throughout the pandemic in Europe, with an estimated 48% of the European population infected.^25^ This could have enhanced the immunity at a different rate for vaccinated and unvaccinated population, leading to an underestimation of VE.^26^

The administration of a second booster for the ≥80 years and other vulnerable population groups in the Spring of 2022 (only in Portugal and Belgium among the participating study sites)^5^ raised VE to around 80% for both hospitalisation and death, and it remained stable between June–July and October–November 2022. However, relative VE did decrease with time since the Spring vaccination campaign and only increased again in October–November 2022, likely reflecting the second booster vaccination rollout in the remaining participating study sites (Appendix S4). Specifically, the second booster was recommended in Summer 2022 (Norway, Belgium [Flanders region]) and in Autumn 2022 (Navarre (Spain), Luxembourg), and Portugal and Belgium introduced the third booster for the ≥80 years in Autumn 2022, resulting in second and third boosters with different vaccine compositions (original strain, Original/Omicron BA.1 and Original/Omicron BA.5) administered simultaneously within and between study sites. The observed similar VE estimates for the second and third boosters in our study suggest that the time since the last dose might be more relevant than the total number of doses received.

In addition, adapted bivalent vaccines were introduced and used as boosters (first, second, third) from September 2022 onwards,^5^ with countries rapidly discontinuing the use of monovalent vaccines. This affects the comparability of the most recent VE estimates with the ones obtained before September 2022 and may have led to the underestimation of the relative benefit of the most recent booster dose. Studies have suggested different effectiveness of monovalent and bivalent vaccines and that bivalent vaccines with BA.4/5 component could provide more protection than those with BA.1.^27^, ^28^

There are several limitations to be flagged. Even though all the sites followed a common protocol, there were some differences in the information available at each site and the outcomes definitions allow a small degree of flexibility. Also, because variables for adjustment collected by study sites were limited by the information available within the respective EHR, there might be some residual confounding in the estimates. The VE monitoring system was implemented in highly vaccinated populations. By October 2021, the primary series vaccination coverage was already >90% in all participating study sites and continued to increase,^4^, ^5^ resulting in a small group of unvaccinated individuals that made VE estimation at the study site level challenging. In particular, at the end of the observation period, this extreme distribution of vaccination led to considerable statistical uncertainty. Henceforth, we envisage that monitoring relative VE, which considers people eligible for the respective booster as a reference group and quantifies the additional benefit of each booster dose, will provide more robust results and will be more informative in the future. Up to March–April 2022, following WHO guidelines we excluded individuals with previous infections, to avoid hybrid immunity effects and increase the internal validity of the estimates. However, a very high incidence was recorded during the Omicron wave at the beginning of 2022, but was not fully captured in EHR used for this study, because systematic testing for SARS‐CoV‐2 was discontinued in most countries and self‐tests were readily available in the community. In this context, the risk of misclassification of previous infection was high. Moreover, in a population where a high proportion has had a previous infection, the generalisability of results obtained using only the fraction without natural exposure to the virus was challenging. Therefore, starting in April–May 2022, the exclusion of people with documented previous infection was no longer applied. This change affects the comparability of the results over time and also might lead to VE underestimation after April 2022.

Sites contributed to the pooled estimates only if a general recommendation of respective doses was in place and if they had registered more than five events in the vaccination groups for each comparison. This results in some estimates excluding particular countries, which could affect the comparability across time. To exclude vulnerable groups with different probabilities of vaccination and developing severe COVID‐19, we excluded those living in long‐term care facilities (if information was available in EHR) and early vaccinees. Our approach might bias VE estimates since vulnerable individuals who remained unvaccinated were not excluded, although primary vaccination coverage in this population was high.

Heterogeneity in VE estimates between study sites was variable, but was often high (Appendix S8, Tables S6–S11). Although the studies were developed using a common protocol, there were some differences in vaccination programs, vaccine brands used, operationalisation of confounding variables and circulating variants between sites that could contribute to this heterogeneity.

While most of the vaccines administered as first, second and third boosters were mRNA vaccines in the EU/EEA, it would be of importance to get brand‐specific estimates. Unfortunately, there was not sufficient information in some registries to provide vaccine brand‐specific estimates. Last but not least, the project aims to expand to additional countries in order to have better geographical representativeness across the EU/EEA.

In conclusion, according to our results, successive COVID‐19 vaccine booster doses have been key to maintaining protection against severe form of the disease over time. Despite the reduction in VE, booster vaccination continues to substantially reduce the risk of hospitalisation and death due to COVID‐19 in older individuals. Overall, this study demonstrated the feasibility of real‐world prospective monitoring of COVID‐19 VE in real time using EHR with the application of a common protocol across six EU/EEA countries. Although it comes with some methodological challenges, the use of population‐based EHR across several sites provide a robust estimate at the EU level and should be maintained to continue with near‐real‐time VE estimates in a changing landscape of COVID‐19 vaccine recommendations.

## AUTHOR CONTRIBUTIONS

The common protocol, including the design of the statistical analysis, was elaborated by SM, IK, BN, AS, JS, MV, AN and NN, with active contributions from IMB, KFN, HM, JC, HDE, CHH, AA, TB, SS, IVE. BS, JAFL, AM, PVB, CD, PH, IC, AE, CB, and AL contributed to common protocol adaptation and site‐specific study implementation. BN, JS, IMB, HM, SS, IK, IVE, and CHH contributed to the data management and analysis at the site level. MM, LA, MD, EK, MFV, and SM were in charge of pooling site estimates. IK and SM drafted the first version of the manuscript. BN, AS, JS, MV, AN, SB, NN, IMB, KFN, HM, JC, HDE, CHH, AA, TB, SS, IVE, BS, JAFL, AM, PVB, CD, PH, IC, AE, CB, AL, MM, LA, MD, EK, and MFV contributed to the interpretation of results and critically reviewed manuscript. All the authors approved the final version of this manuscript.

## CONFLICT OF INTEREST STATEMENT

The authors declare no conflict of interest.

### PEER REVIEW

The peer review history for this article is available at https://www.webofscience.com/api/gateway/wos/peer-review/10.1111/irv.13195.

## ETHICS STATEMENT

All participating study sites conformed with their respective national and EU ethical and data protection requirements. Ethical statements for each of the participating study sites are reported in Supporting Information (Appendix S3).

## Supporting information


**Appendix S1.** Data sources used in the six study sites to extract the study variables.
**Appendix S2.** Methodological details in the different study sites.
**Appendix S3.** Ethical statements for six study sites.
**Appendix S4.** Age‐specific rollout of the COVID‐19 vaccination campaign for the 65–79 year‐olds and ≥80‐year‐olds by vaccine dose and study site.
**Appendix S5.** Number of events and person‐months for VE estimates against hospitalisation and death due to COVID‐19.
**Appendix S6.** Detailed results of vaccine effectiveness against hospitalisation due to COVID‐19.
**Appendix S7.** Detailed results of vaccine effectiveness against death due to COVID‐19.
**Appendix S8.** Measures of heterogeneity from the random‐effects meta‐analysis pooling site‐specific estimates from six EU/EEA countries.

## Data Availability

The data that support the findings of this study were made available under a license that the authors do not have permission to share due to privacy and ethical restrictions. Requests to access the raw data should be directed to the data owners of electronic health registries at each study site.

## References

[1] European Medicines Agency . COVID‐19 Vaccines: Authorised. EMA; 2021.

[2] Machado A , Kislaya I , Rodrigues AP , et al. COVID‐19 vaccine effectiveness against symptomatic SARS‐CoV‐2 infections, COVID‐19 related hospitalizations and deaths, among individuals aged ≥65 years in Portugal: a cohort study based on data‐linkage of national registries February‐September 2021. PLoS One [Internet]. 2022;17(9 September):e0274008. doi:10.1371/journal.pone.0274008 36099273 PMC9469958

[3] Gram MA , Emborg HD , Schelde AB , et al. Vaccine effectiveness against SARS‐CoV‐2 infection or COVID‐19 hospitalization with the Alpha, Delta, or Omicron SARS‐CoV‐2 variant: a nationwide Danish cohort study. PLoS Med. 2022;19(9):e1003992. doi:10.1371/journal.pmed.1003992 36048766 PMC9436060

[4] European Centre for Disease Prevention and Control (ECDC) . COVID‐19 Vaccine Tracker [Internet]. [cited 2022 Feb 6]. Available from: https://vaccinetracker.ecdc.europa.eu/public/extensions/COVID-19/vaccine-tracker.html#uptake-tab

[5] ECDC . Overview of the implementation of COVID‐19 vaccination strategies and vaccine deployment plans in the EU/EEA: April 2022. ECDC Technical Report. 2022.

[6] Feikin DR , Higdon MM , Andrews N , et al. Assessing COVID‐19 vaccine effectiveness against Omicron subvariants: report from a meeting of the World Health Organization. Vaccine. 2023;41(14):2329‐2338. doi:10.1016/j.vaccine.2023.02.020 36797097 PMC9910025

[7] Björk J , Bonander C , Moghaddassi M , et al. COVID‐19 vaccine effectiveness against severe disease from SARS‐CoV‐2 Omicron BA.1 and BA.2 subvariants—surveillance results from southern Sweden, December 2021 to March 2022. Eurosurveillance. 2022;27(18):2200322. 35514304 10.2807/1560-7917.ES.2022.27.18.2200322 PMC9074397

[8] Tartof SY , Slezak JM , Puzniak L , et al. BNT162b2 vaccine effectiveness against SARS‐CoV‐2 omicron BA.4 and BA.5. Lancet Infect Dis. 2022;22(12):1663‐1665. doi:10.1016/S1473-3099(22)00692-2 PMC9597567 36306800

[9] Meurisse M , Catteau L , van Loenhout JAF , et al. Homologous and heterologous prime‐boost vaccination: impact on clinical severity of SARS‐CoV‐2 omicron infection among hospitalized COVID‐19 patients in Belgium. Vaccines (Basel). 2023;11(2):378. doi:10.3390/vaccines11020378 36851257 PMC9961733

[10] Martínez‐Baz I , Miqueleiz A , Casado I , et al. Effectiveness of COVID‐19 vaccines in preventing SARS‐CoV‐2 infection and hospitalisation, Navarre, Spain. Eurosurveillance. 2021;26(21):2100438. doi:10.2807/1560-7917.ES.2021.26.21.2100438 34047271 PMC8161727

[11] Starrfelt J , Danielsen AS , Buanes EA , et al. Age and product dependent vaccine effectiveness against SARS‐CoV‐2 infection and hospitalisation among adults in Norway: a national cohort study, July–November 2021. BMC Med. 2022;20(1):1‐11. Available from: https://pubmed.ncbi.nlm.nih.gov/36050718/ 36050718 10.1186/s12916-022-02480-4 PMC9436448

[12] Andrews N , Stowe J , Kirsebom F , et al. Effectiveness of COVID‐19 booster vaccines against covid‐19 related symptoms, hospitalisation and death in England. Nat Med. 2022;24(4):831‐837. doi:10.1038/d41591-022-00013-3 PMC9018410 35045566

[13] Arbel R , Sergienko R , Friger M , et al. Effectiveness of a second BNT162b2 booster vaccine against hospitalization and death from COVID‐19 in adults aged over 60 years. Nat Med. 2022;28(7):1486‐1490. doi:10.1038/s41591-022-01832-0 35468276

[14] European Centre for Disease Prevention and Control . Protocol for a COVID‐19 vaccine effectiveness study using health data registries. 2023.

[15] Direction de la santé . Rapport sur l'effectivité vaccinale contre la COVID‐19 au Luxembourg. 2021.

[16] Schmidt M , Pedersen L , Sørensen HT . The Danish Civil Registration System as a tool in epidemiology. Eur J Epidemiol. 2014;29(8):541‐549. doi:10.1007/s10654-014-9930-3 24965263

[17] Sentís A , Kislaya I , Nicolay N , et al. Estimation of COVID‐19 vaccine effectiveness against hospitalisation in individuals aged ≥ 65 years using electronic health registries; a pilot study in four EU/EEA countries, October 2021 to March 2022. Euro Surveill. 2022;27(30):2200551. doi:10.2807/1560-7917.ES.2022.27.30.2200551 35904059 PMC9336167

[18] World Health Organization (WHO) . WHO COVID‐19: Case Definitions. Updated in Public health surveillance for COVID‐19.

[19] ECDC . Case definition for COVID‐19, as of 3 December 2020 [Internet]. [cited 2023 Mar 15]. Available from: https://www.ecdc.europa.eu/en/covid-19/surveillance/case-definition

[20] Deeks JJ , Higgins JPT , Altman DG . Chapter 10: Analysing data and undertaking meta‐analyses. In: Higgins JPT , Thomas J , Chandler J , et al., eds. Cochrane Handbook for Systematic Reviews of Interventions Version 63; 2022.

[21] European Centre for Disease Prevention and Control . Data on SARS‐CoV‐2 variants in the EU/EEA [Internet]. 2022 [cited 2023 Mar 6]. Available from: https://www.ecdc.europa.eu/en/publications-data/data-virus-variants-covid-19-eueea

[22] Cele S , Jackson L , Khoury DS , et al. Omicron extensively but incompletely escapes Pfizer BNT162b2 neutralization. Nature. 2022;602(7898):654‐656. doi:10.1038/s41586-021-04387-1 35016196 PMC8866126

[23] Hachmann NP , Miller J , Collier A , et al. Neutralization escape by SARS‐CoV‐2 Omicron subvariants BA.2.12.1, BA.4, and BA.5. New Engl J Med. 2022;387(1):86‐88. doi:10.1056/NEJMc2206576 35731894 PMC9258748

[24] Tenforde MW , Self WH , Gaglani M , et al. Effectiveness of mRNA vaccination in preventing COVID‐19–associated invasive mechanical ventilation and death—United States, March 2021–January 2022. MMWR Morb Mortal Wkly Rep. 2022;71(12):459‐465. doi:10.15585/mmwr.mm7112e1 35324878 PMC8956334

[25] Bergeri I , Whelan MG , Ware H , et al. Global SARS‐CoV‐2 seroprevalence from January 2020 to April 2022: a systematic review and meta‐analysis of standardized population‐based studies. PLoS Med. 2022;19(11):e1004107. doi:10.1371/journal.pmed.1004107 36355774 PMC9648705

[26] Halloran ME , Longini IM , Struchiner C . Design and Analysis of Vaccine Studies.

[27] Lin DY , Xu Y , Gu Y , et al. Effectiveness of bivalent boosters against severe omicron infection. New Engl J Med. 2023;388(8):764‐766. doi:10.1056/NEJMc2215471 36734847 PMC9933929

[28] Worm Andersson N , Thiesson EM , Baum U , Pihlström N , Starrfelt J , Faksová K , et al. Comparative effectiveness of the bivalent BA.4–5 and BA.1 mRNA‐booster vaccines in the Nordic countries. 10.1136/bmj-2022-075286 PMC10364194 37491022

