# Supplementary material for: Monitoring COVID‐19 vaccine effectiveness against COVID‐19 hospitalisation and death using electronic health registries in ≥65 years old population in six European countries, October 2021 to November 2022
Source: Influenza Other Respir Viruses. 2023 Nov 28;17(11):e13195. doi: 10.1111/irv.13195 (PMC10682901; doi:10.1111/irv.13195)
Supplement: Supplementary file 1 — Appendix S1. Data sources used in the six study sites to extract the study variables. Appendix S2. Methodological details in the different study sites. Appendix S3. Ethical statements for six study sites. Appendix S4. Age‐specific rollout of the COVID‐19 vaccination campaign for the 65–79 year‐olds and ≥80‐year‐olds by vaccine dose and study site. Appendix S5. Number of events and person‐months for VE estimates against hospitalisation and death due to COVID‐19. Appendix S6. Detailed results of vaccine effectiveness against hospitalisation due to COVID‐19. Appendix S7. Detailed results of vaccine effectiveness against death due to COVID‐19. Appendix S8. Measures of heterogeneity from the random‐effects meta‐analysis pooling site‐specific estimates from six EU/EEA countries. [file IRV-17-e13195-s001.docx]

**Supplementary material**

**Monitoring COVID-19 vaccine effectiveness against hospitalisation and death using electronic health registries in ≥65 years old population in six European countries, October 2021 to November 2022**

Irina Kislaya, Alexis Sentís, Jostein Starrfelt, Baltazar Nunes, Iván Martínez-Baz, Katrine Finderup Nielsen, Ala'a Al Kerwi, Toon Braeye, Mario Fontán, Hinta Meijerink, Jesús Castilla, Hanne-Dorthe Emborg, Christian Holm Hansen, Susanne Schmitz, Izaak Van Evercooren, Marta Valenciano, Anthony Nardone, Nathalie Nicolay, Susana Monge, VEBIS-Lot4 working group

**Table of contents**

[Appendix 1. Data sources used in the six study sites to extract the study variables 2](#_Toc142915805)

[Appendix 2. Methodological details in the different study sites 5](#_Toc142915806)

[Appendix 3. Ethical statements for six study sites 9](#_Toc142915807)

[Appendix 4. Age-specific rollout of the COVID-19 vaccination campaign for the 65-79 year-olds and ≥80-year-olds by vaccine dose and study site 10](#_Toc142915808)

[Appendix 5. Number of events and person-months for VE estimates against hospitalisation and death due to COVID-19 11](#_Toc142915809)

[Appendix 6. Detailed results of vaccine effectiveness against hospitalisation due to COVID-19 13](#_Toc142915810)

[Appendix 7. Detailed results of vaccine effectiveness against death due to COVID-19 15](#_Toc142915811)

[Appendix 8. Measures of heterogeneity from the random-effects meta-analysis pooling site-specific estimates from six EU/EEA countries 20](#_Toc142915812)

## Appendix 1. Data sources used in the six study sites to extract the study variables

| **Type of variables** | **Study variable** | **Study site** | | | | | |
| --- | --- | --- | --- | --- | --- | --- | --- |
|  |  | **Portugal** | **Navarre (Spain)** | **Norway*** | **Denmark** | **Luxembourg** | **Belgium** |
| **Outcomes** | **Hospital admission due to COVID-19** | National Hospital Discharge database (BIMH) | Enhanced COVID surveillance with individual revision of events | Norwegian Intensive Care and Pandemic Registry (NIPaR) | Danish National Patient Register (DNPR) | Epidemiological national surveillance platform (MSINF) to collect daily data from hospitals | Clinical Hospital Survey database |
|  | **Death due to COVID-19** | National Death Registry (SICO) and National Health Service User databaset (NHSU): a Cause of death is from SICO, death status and date of death from NHSU. | Administrative database of deaths and individual revision of events | Norwegian Death Registry (DÅR) | MiBA and Danish Civil Registration system (CPR) | Idem + death certificate for death happened outside hospital or nursing home | NA |
| **Exposures** | **Vaccination status** | The National Vaccination Register (VACINAS) | Vaccination register | The National Immunisation Register (SYSVAK) | Danish Vaccination Registry (DVR) | MSVAC: National vaccination registry under the responsibility of Health Directorate | National vaccine registry (VACCINNET) |
| **Variables for adjustment or stratification** | **Age** | National Health Service User databaset (NHSU) | Administrative database | The National Population Register (Folkeregisteret) | CPR | Statutory health insurance database | The national population register |
|  | **Sex** | National Health Service User databaset (NHSU) | Administrative database | The National Population Register (Folkeregisteret) | CPR | Statutory health insurance database | national population register |
|  | **Health Region** | Region of residence: National Health Service User databaset (NHSU) | Not applicale | County of residence at end of study period: The National Population Register (Folkeregisteret) | CPR | Statutory health insurance database | Province of residence:  national population register |
|  | **Comorbidities** | Primary Care Information System (SIM@SNS). | Administrative database | Risk groups / Comorbidities: Based on Norwegian Patient Registry (NPR) | DNPR | NA | Intermutualistic Agency database |
|  | **Previous infection** | National Information System for Epidemiologic Surveillance (BI-SINAVE) | Previos infections are excluded, pendent of a sepatare analysis | The Surveillance System for Infectious Diseases (MSIS) | MiBA | MSINF (see above) | COVID-19 Laboratory test results database from Healthdata.be register |
|  | **Others specific to the study site** | 1. Number of tests for SARS-CoV-2 in 2020-2022: BI-SINAVE  2. Conditions of living – Deprivation at municipality level: Most recent data from 2011  3. Other vaccines uptake: VACINAS | 1. Country of birth and high functional dependence:  Administrative database | 1. Conditions of living – Crowding: Statistics Norway (SSB). Most recent data from 2019 – separate level for missing data  2. *County of birth:* Folkeregisteret | NA | 1. Statutory health insurance database | 1. Household income (according to tax records) categorized as low (lowest 40%), mid (middle 30%), and high (highest 30%):  STATBEL database |
|  |  |  |  |  |  |  |  |

*****  All data in Nowray was integrated in the emergency preparedness register for COVID-19 (Beredt C19), https://www.fhi.no/en/id/infectious-diseases/coronavirus/emergency-preparedness-register-for-covid-19/

## Appendix 2. Methodological details in the different study sites

| **Variable** | **Definition, categorisation, use in the model** | | | | | |
| --- | --- | --- | --- | --- | --- | --- |
|  | **Portugal** | **Navarre (Spain)** | **Norway** | **Denmark** | **Luxembourg** | **Belgium** |
| **Age** | Age at the start of study period (5-year categories) | Age at the start of study period (5-year categories) | Age at end of 2022 (birth cohorts) (For adjustment: 5-year age groups) | 5-17, 18-49, 50-64, 65-79, 80+, adjusted in bins: 5-9, 10-14, 15-17, 18-24 and then 5-year bins until the final category, 90+ years | Age at the start of follow up, 5-year bins | Age in years at the end of the year in which the study period begins.  For adjustment: 5-year age groups. |
| **Comorbidities** | Number of comorbidities (0, 1, 2, 3, 4, 5+)  *Considered comorbidities include: anemia, asthma, cancer, cardiac disease, dementia, diabetes, hypertension, HIV, liver disease, neuromuscular disease, obesity, pulmonary disease, renal disease, rheumatologic disease, stroke, tuberculosis* | Immunocompromised  Other major chronic conditions,  High functional dependencies | High risk:   - *Organ transplant* - *Immunodeficiency* - *Haematological cancer in the last five years* - *Other active cancers* - *Neurological or neuromuscular diseases that cause impaired cough or lung function (e.g., ALS and cerebral palsy)* - *Chronic kidney disease, or significant renal impairment.*   Medium risk:   - *Chronic liver disease or significant hepatic impairment* - *Immunosuppressive therapy* - *Diabetes* - *Chronic lung disease including cystic fibrosis and severe asthma which have required the use of high dose inhaled or oral steroids within the past year* - *Obesity with a body mass index (BMI) of ≥35 kg/m2* - *Dementia* - *Chronic heart and vascular disease (with the exception of high blood pressure) and stroke* | Immunocompromised, including:   - *HIV* - *Immunological disease* - *Radiation therapy* - *Organtransplanted*   Other, including:   - *Diabetes* - *Obesity* - *Cancer* - *Neurological Disease* - *Kidney disease* - *Haematological cancers* - *Heart disease* - *Chronic respiratory disease* - *Liver disease (incl. alcohol lever)* - *Endocrine Disease* - *Hematological Disease* - *Coagulation Disease* - *Innate Diseases* - *TB* - *Missing a lung* - *Missing a kidney* | Not included | No comorbidities associated with an increased risk for severe COVID-19 infection.  At least one comorbidity which increases the risk for severe COVID-19 infection and not being immunocompromised (medium risk):   - *Received chemotherapy/ radiotherapy against cancer* - *Received multidisciplinary oncologic consult* - *Cardiovascular illness – general* - *Cardiovascular illness- specifically a heart disease* - *Alzheimer* - *Asthma* - *Haemophilia* - *Disease of Crohn, Colitis Ulcerosa, Psoriatrische arthritis, Reumatoid arthritis* - *Chronic obstructive pulmonary disease* - *Diabetes with cardiovascular compilations* - *Diabetes Mellitus with insulin treatment* - *Epilepsy and neuropathic pain* - *Chronic hepatitis type B or C* - *Kidney failure* - *Cystic fibrosis* - *Exocrine pancreatic disease* - *Disease of Parkinson* - *Psoriasis* - *Psychosis occurring with people older than 70 years* - *Psychosis occurring with people of 70 year or younger.* - *Multiple sclerosis* - *Thrombosis while treated with antithrombotic medicines* - *Thyroid disorder* - *HIV*   Immunocompromised (high risk): *if a person received a priority invitation for a COVID-19 vaccination due to being immunocompromised, then he/she was classified into a this group.* |
| **Country of residence / country of birth / nationality** | Not included | Country of birth | As registered at time of analysis (June 2022) | Not included | Country of residence = administrative address in Luxembourg (as of September 2021)  Country of birth = Luxembourg / Other  Nationality = Citizenship Luxembourg / Other | Not included |
| **Deprivation index or similar** | European deprivation index quintile Q1 (least deprived) to Q5 (most deprived) |  | Crowded conditions: if the number of rooms is lower than the number of residents or one resident lives in one room, and the number of square metres (P-area) is below 25 sq. m. per person. If the number of rooms or the P-area is not specified, a household will be regarded as crowded if one of these criteria is met (incomplete and slightly outdated data) | Not included | Not included | Household income: low (lowest 40%)-medium (middle 30%)-high (highest 30%) |
| **Geographic level** | Region of residence (North, Center, Lisbon and Tagus Valey, Alentejo, Algarve) | Not included | County of residence | Adjustment for residency in the 5 geographical regions of Denmark (EU NUTS-2 regions) | Canton | Province of residence |
| **Other vaccines uptake** | Vaccination against influenza, PCV7, PCV10, PCV13 or PPV23 in the last 3 years | Not included | Not included | Not included | Not included | Not included |
| **Number of COVID-19 tests in 2020-2022** | 0, 1, 2, 3, 4-9, 10+ | Not included | Not included | Positive RT-PCR test for SARS-CoV-2 | Not included | Not included |

## Appendix 3. Ethical statements for six study sites

All study sites participating in this study conformed with their respective national and EU ethical and data protection requirements.

Ethical statements for each of the participating study sites:

**Belgium:** Data linkage and collection within the data-warehouse have been approved by the information security committee. The study was conducted in accordance with the Declaration of Helsinki. Ethical approval was granted for the gathering of data from hospitalized patients by the Committee for Medical Ethics from the Ghent University Hospital (reference number BC-07507) and authorization for possible individual data linkage using the national register number from the Information Security Committee (ISC) Social Security and Health (reference number IVC/KSZG/20/384). Linkage of hospitalized patient data to vaccination and testing within the LINK-VACC project was approved by the Medical Ethics Committee UZ Brussels–VUB on 3 February 2021 (reference number 2020/523), and authorization from the ISC Social Security and Health (reference number IVC/KSZG/21/034).

**Denmark**: We used only administrative register data for the study. According to Danish law, ethics approval is exempt for such research, and the Danish Data Protection Agency, which is dedicated ethics and legal oversight body, thus waives ethical approval for our study of administrative register data when no individual contact of participants is necessary, and only aggregate results are included as findings. The study is, therefore, fully compliant with all legal and ethical requirements, and there are no further processes available regarding such studies.

**Luxembourg**: The planning, conduct and reporting of the study are in line with the Declaration of Helsinki. Official ethical approval and patients’ consent are not required, as data collection is part of the national pandemic surveillance system set-up under the authority of the Ministry of Health.

**Navarre (Spain)**: The study was approved by Navarre’s Ethical Committee for Clinical Research, which waived the requirement of obtaining informed consent.

**Norway**: Ethical approval was granted by Regional Committees for Medical and Health Research Ethics (REC) Southeast (reference number 122745). The Norwegian Institute of Public Health has performed a Data Protection Impact Assessment (DPIA) for Beredt C19.

**Portugal**: The study received approval from the Ethical Committee and the Data Protection Officer of the Instituto Nacional de Saúde Doutor Ricardo Jorge. Given that data was irreversibly anonymised, the need for the participants’ informed consent was waived by the Ethical Committee.

## Appendix 4. Age-specific rollout of the COVID-19 vaccination campaign for the 65-79 year-olds and ≥80-year-olds by vaccine dose and study site

|  | ≥80-year-olds | | | | 65-79-year-olds | | | |
| --- | --- | --- | --- | --- | --- | --- | --- | --- |
|  | Primary course | 1st booster | 2nd booster | 3rd booster | Primary course | 1st booster | 2nd booster | 3rd booster |
| Belgium | 2021.03.05 | 2021.09.22 | 2022.07.20 | 2022.09.12 | 2021.03.08 | 2021.09.22 | 2022.09.12 |  |
| Denmark | 2020.12.28 | 2021.10.18 | 2022.09.15 (85+) |  | 2021.03.15 | 2021.10.18 | 2022.10.01 |  |
| Luxembourg | 2021.01.27 | 2021.07.07 | 2022.04.12 |  | 2021.01.27  (75+) | 2021.11.08 | 2022.07.14 |  |
| Navarre (Spain) | 2021.03.02 | 2021.10.25 | 2022.10.10 |  | 2021.04.09 | 2021.10.25 | 2022.10.10 |  |
| Norway | 2021.01.31 | 2021.10.05 | 2022.06.29 | n.a. | 2021.03.07 | 2021.10.05 | 2022.06.29 | n.a. |
| Portugal | 2021.02.03 | 2021.10.11 | 2022.05.16 | 2022.09.06 | 2021.03.30 | 2021.10.11 | 2022.09.06 |  |

## Appendix 5. Number of events and person-months for VE estimates against hospitalisation and death due to COVID-19

**Table S1**. Number of hospitalizations due to COVID-19, and person-months for estimates of vaccine effectiveness (VE) against hospitalisation by vaccination status and age, in overlapping eight-week wide observation intervals from October 2021 to November 2022 in six EU/EEA countries.

| **Vaccination status** | **Age group** | **Follow-up period** | | | | | | | | | | | | |
| --- | --- | --- | --- | --- | --- | --- | --- | --- | --- | --- | --- | --- | --- | --- |
|  |  | October 1 to November 25, 2021^‡^ | November 1 to December 26, 2021^‡^ | December 1, 2021 to January 25, 2022^‡^ | January 1 to February 25, 2022^‡^ | February 1 to March 28, 2022^‡^ | March 1 to April 25, 2022^‡^ | April 1 to May 26, 2022^‡^ | May 1 to June 25, 2022^‡^ | June 1 to July 26, 2022 | July 1 to August 25, 2022 | August 1 to September 25, 2022 | September 1 to October 26, 2022 | October 1, 2022 to November 25, 2022 |
| **Unvaccinated**^†^ | 65-79-year-olds | 229/  311,465 | 463/ 295,571 | 535/  280,857 | 463/  269,006 | 370/  257,793 | 208/  263,817 | 105/  285,486 | 100/  291,490 | 111/  287,689 | 105/  289,087 | 61/  289,785 | 71/  294,033 | 60/  290,185 |
|  | 80+ year-olds | 130/  147,142 | 275/ 140,295 | 453/  135,547 | 546/  129,959 | 443/  125,031 | 260/  122,399 | 195/  127,395 | 204/  128,457 | 182/  125,213 | 150/  118,527 | 103/  118,803 | 92/  136,477 | 99/  117,273 |
| **Complete primary vaccination** | 65-79-year-olds | 769/ 5,430,697 | 1141/ 3,627,044 | 766/ 1,261,165 | 515/ 386,221 | 340/ 266,654 | 192/ 237,183 | 143/ 458,050 | 126/ 358,105 | 108/ 373,015) | 108/ 391,304 | 61/ 374,079 | 48/ 344,104 | 39/ 239,640 |
|  | 80+ year-olds | 652/ 1,561,711 | 751/ 716,577 | 620/ 262,138 | 544/ 148,922 | 399/ 117,721 | 238/ 106,890 | 214/ 151,184 | 237/ 129,272 | 187/ 117,594 | 132/ 117,325 | 64/ 110,983 | 68/ 98,404 | 65/ 89,648) |
| **Complete primary vaccination + first booster** | 65-79-year-olds | 47/ 104,549 | 169/ 1,199,370 | 597/ 3,623,380 | 1,429/ 4,981,833 | 2,001/ 4,906,390 | 1,207/ 4,600,781 | 920/ 4,971,604 | 928/ 5,052,588 | 1,282/ 4,935,561 | 1,180/ 5,298,858 | 707/ 5,140,450 | 635/ 4,161,751 | 376/ 2,551,844 |
|  | 80+ year-olds | 38/ 308,135 | 181/ 1,124,695) | 884/ 1,752,544 | 1,938/ 1,901,312 | 2,541/ 1,871,887 | 1,910/ 1,759,710 | 1,762/ 1,843,384 | 1,547/ 1,175,616 | 1,578/ 1,275,533 | 1,345/ 1,273,692 | 759/ 1,151,542 | 597/ 794,254 | 379/ 515,988 |
| **Complete primary vaccination + second booster** | 65-79-year-olds | N/A | N/A | N/A | N/A | N/A | N/A | N/A | N/A | N/A | N/A | 68/ 195,897 | 71/ 528,062 | 56/ 1,505,538 |
|  | 80+ year-olds | N/A | N/A | N/A | N/A | N/A | N/A | N/A | N/A | 123/ 425,531 | 224/ 657,717 | 191/ 728,417 | 209/ 677,500 | 144/ 641,448 |

N/A = Not applicable

^†^ Unvaccinated individuals are only captured in five countries (all except Belgium).

‡ Results up to June 1 to July 26, 2022 are limited to four sites: Denmark, Navarre (Spain), Norway and Portugal

**Table S2**. Number of COVID-19 related deaths, and person-months for estimates of vaccine effectiveness (VE) against COVID-19 related death by vaccination status and age, in overlapping eight-week wide observation intervals from October 2021 to November 2022 in five^†^ EU/EEA countries

| **Vaccination status** | **Age group** | **Follow-up period** | | | | | | | | | | | | |
| --- | --- | --- | --- | --- | --- | --- | --- | --- | --- | --- | --- | --- | --- | --- |
|  |  | October 1 to November 25, 2021^‡^ | November 1 to December 26, 2021^‡^ | December 1, 2021 to January 25, 2022^‡^ | January 1 to February 25, 2022^‡^ | February 1 to March 28, 2022^‡^ | March 1 to April 25, 2022^‡^ | April 1 to May 26, 2022^‡^ | May 1 to June 25, 2022^‡^ | June 1 to July 26, 2022 | July 1 to August 25, 2022 | August 1 to September 25, 2022 | September 1 to October 26, 2022 | October 1, 2022 to November 25, 2022 |
| **Unvaccinated** | 65-79-year-olds | N/A | N/A | N/A | N/A | N/A | 85/  263,853 | 57/  278,851 | 52/  284,851 | 51/  281,009 | 30/  282,408 | 30/  282,976 | 32/  278,937 | 21/  282,612 |
|  | 80+ year-olds | N/A | N/A | N/A | N/A | N/A | 225/  122,428 | 164/  127,799 | 211/  128,885 | 188/  125,635 | 93/  118,958 | 76/  119,255 | 88/  136,915 | 77/  117,730 |
| **Complete primary vaccination** | 65-79-year-olds | N/A | N/A | N/A | N/A | N/A | 102/ 232,313 | 100/ 382,715 | 96/ 352,210 | 68/ 367,132 | 44/ 379,412 | 26/ 375,545 | 15/ 251,195 | 20/ 210,453 |
|  | 80+ year-olds | N/A | N/A | N/A | N/A | N/A | 212/ 105,385 | 241/ 151,996 | 276/ 131,997 | 204/ 118,401 | 102/ 116,330 | 61/ 111,688 | 56/ 99,027 | 47/ 73,714 |
| **Complete primary vaccination + first booster** | 65-79-year-olds | N/A | N/A | N/A | N/A | N/A | 421/ 4,467,690) | 451/ 4,831,083 | 499/ 4,913,682 | 412/ 4,797,355 | 323/ 5,091,730 | 244/ 4,941,744 | 243/ 3,968,103 | 174/ 2,438,372 |
|  | 80+ year-olds | N/A | N/A | N/A | N/A | N/A | 1,258/ 1,759,883 | 1,437/ 1,845,307 | 1,480/ 1,636,612 | 923/ 1,277,984 | 600/ 1,276,266 | 313/ 1,093,178 | 268/ 785,472 | 228/ 464,819 |
| **Complete primary vaccination + second booster** | 65-79-year-olds | N/A | N/A | N/A | N/A | N/A | N/A | N/A | N/A | N/A | N/A | N/A | N/A | 37/ 768,103 |
|  | 80+ year-olds | N/A | N/A | N/A | N/A | N/A | N/A | N/A | N/A | 247/ 425,607 | 227/ 658,215 | 190/ 729,420 | 174/ 660,644 | 153/ 388,747 |

N/A = Not applicable

† Belgium did not provide estimates on COVID-19 related death and did not participate in this analysis.

‡ Results up to June 1 to July 26, 2022 are limited to four sites: Denmark, Navarre (Spain), Norway and Portugal

## Appendix 6. Detailed results of vaccine effectiveness against hospitalisation due to COVID-19

**Figure S1.** Pooled booster dose(s) relative vaccine effectiveness (vs. primary vaccination≥169 days ago) against COVID-19 hospitalisation by age group, in overlapping eight-week wide observation intervals from April 2022 to November 2022, in six EU/EEA countries. Random effects meta-analysis.


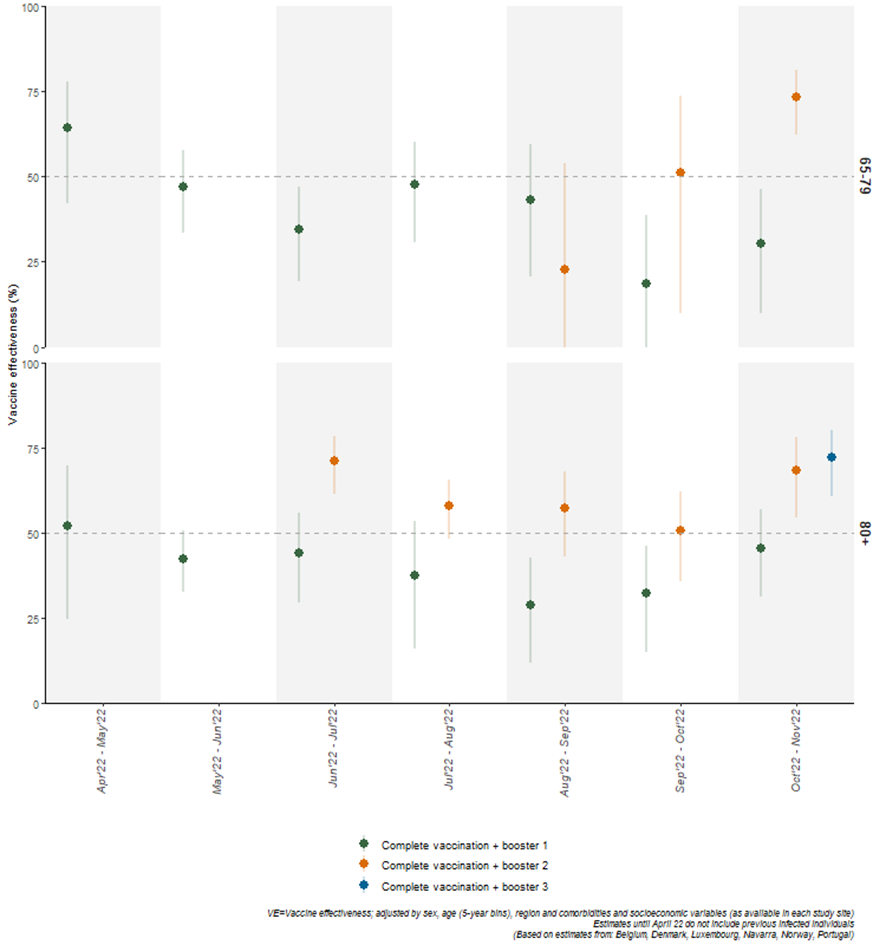


**Figure S2.** Pooled booster dose(s) relative VE (vs. one less dose) against COVID-19 hospitalisation by age group, in overlapping eight-week wide observation intervals from April 2022 to November 2022, in six EU/EEA countries. Random effects meta-analysis.


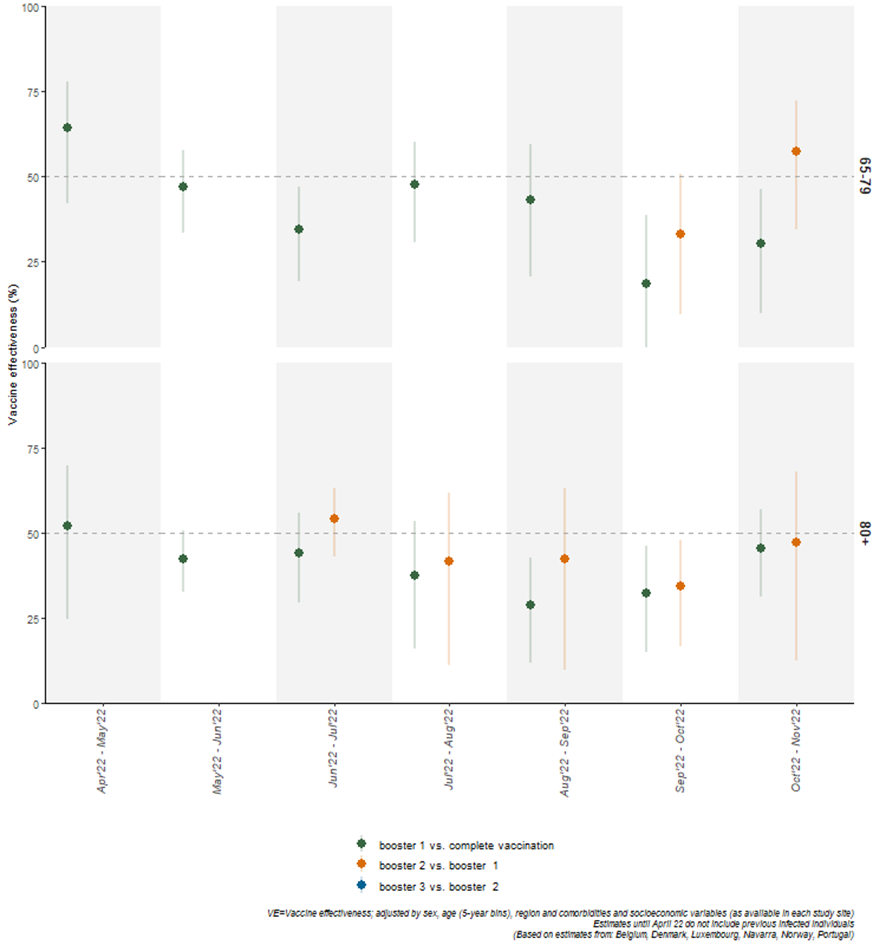


## Appendix 7. Detailed results of vaccine effectiveness against death due to COVID-19

**Figure S3.** Pooled booster dose(s) relative VE (vs. complete vaccination≥169 days ago) against COVID-19 death by age group, in overlapping eight-week wide observation intervals from April 2022 to November 2022, in five EU/EEA countries. Random effects meta-analysis.

**
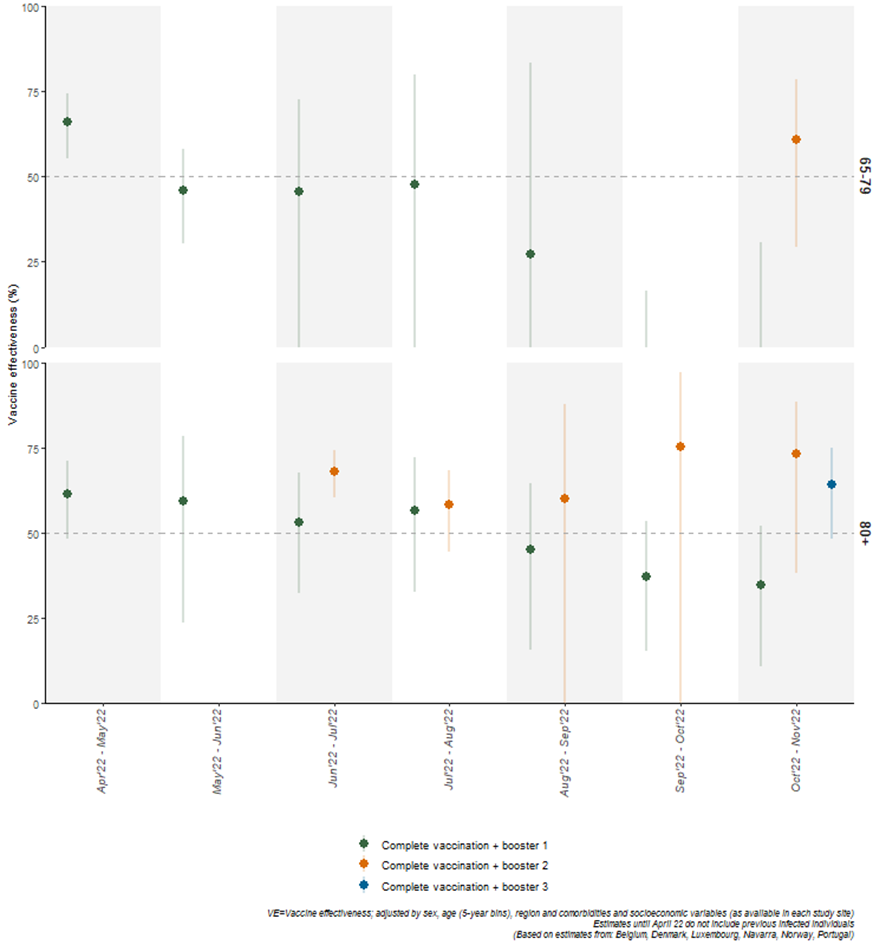
**

**Figure S4.** Pooled booster dose(s) relative VE (vs. one less dose) against COVID-19 death by age group, in overlapping eight-week wide observation intervals from April 2022 to November 2022, in five EU/EEA countries. Random effects meta-analysis.


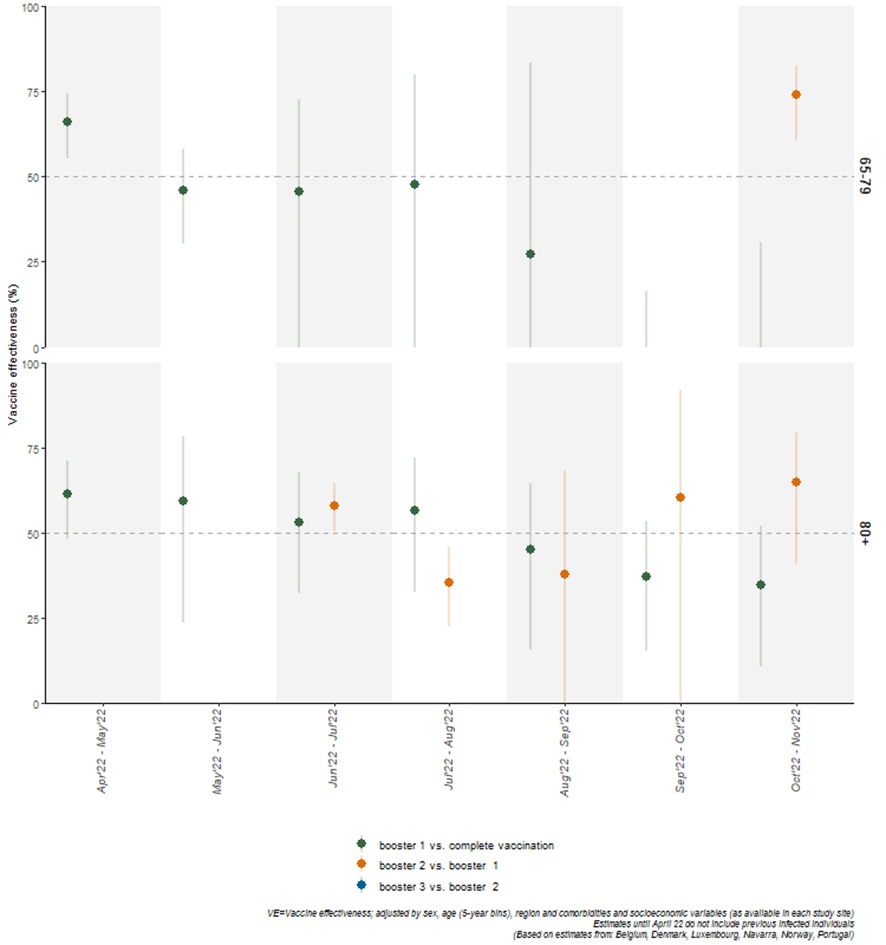


**Table S3**. Estimated vaccine effectiveness (VE) for primary vaccination (vs. unvaccinated) against COVID-19 death by age group, in overlapping eight-week wide observation intervals from March 2022 to November 2022, in five^†^ EU/EEA countries. Random effects meta-analysis.

| **Age group** | **VE (95% CI) vs.unvaccinated** | | | | | | | | | | | | | |
| --- | --- | --- | --- | --- | --- | --- | --- | --- | --- | --- | --- | --- | --- | --- |
|  | October 1 to November 25, 2021^‡^ | November 1 to December 26, 2021^‡^ | December 1, 2021 to January 25, 2022^‡^ | January 1 to February 25, 2022‡ | February 1 to March 28, 2022^‡^ | March 1 to April 25, 2022^‡^ | April 1 to May 26, 2022^‡^ | May 1 to June 25, 2022^‡^ | June 1 to July 26, 2022^‡^ | July 1 to August 25, 2022 | August 1 to September 25, 2022 | September 1 to October 26, 2022 | October 1, 2022 to November 25, 2022 |  |
| 65-79-year-olds | N/A | N/A | N/A | N/A | N/A | 21.3%  (-19.8; 48.2) ^§^ | 48.4%  (1.3; 73) ^§^ | 56.8%  (37.1; 70.3) ^§^ | 55.3% (33.9; 69.9) ^§^ | 46.1%  (-12.4; 74.1) ^§¶^ | 66.2%  (-21.5; 90.6) ^§¶^ | 75.0%  (48.0; 88.0) ^§¶#◊^ | 45.0%  (-28; 76.4) ^§¶#◊^ |  |
| 80+ year-olds | N/A | N/A | N/A | N/A | N/A | 41.4%  (26; 53.6) ^§^ | 45.1%  (-16.5; 74.2) ^§^ | 17.6%  (-281.6; 82.2) ^§^ | 32.5%  (-14.6; 60.2) | 28.7%  (-71.9; 70.4) ^§¶^ | 61.9% (45.6; 73.3) ^§¶^ | 54.3% (23.9; 72.6) ^§¶^ | 45.1%  (-29.2; 76.6) ^§¶◊^ |  |

VE = Vaccine effectiveness; CI = confidence interval; N/A: Not applicable: In the first six months of the study VE estimates were not available for death outcome

^†^ Unless otherwise indicated, results are based on pooling VE estimates from Denmark, Luxembourg, Navarre (Spain), Norway and Portugal. (Belgium did not provide estimates of VE against COVID-10 death).

‡ Unless otherwise indicated, results up to June 1 to July 26, 2022 are limited to four sites: Denmark, Navarre (Spain), Norway and Portugal

^§^ Navarre (Spain) did not reach 5 events or this dose was still not recommended at this site and did not contribute to the estimate

^¶^ Luxembourg did not reach 5 events or this dose was still not recommended at this site and did not contribute to the estimate

^#^ Denmark did not reach 5 events or this dose was still not recommended at this site and did not contribute to the estimate

^◊^ Norway did not reach 5 events or this dose was still not recommended at this site and did not contribute to the estimate

**Table S4**. Estimated vaccine effectiveness (VE) and relative vaccine effectiveness (rVE) for the first booster dose against COVID-19 death by age group, in overlapping eight-week wide observation intervals from October 2021 to November 2022, in five^†^ EU/EEA countries. Random effects meta-analysis.

| **Age group** | October 1 to November 25, 2021^‡^ | November 1 to December 26, 2021^‡^ | December 1, 2021 to January 25, 2022^‡^ | January 1 to February 25, 2022^‡^ | February 1 to March 28, 2022^‡^ | March 1 to April 25, 2022^‡^ | April 1 to May 26, 2022^‡^ | May 1 to June 25, 2022^‡^ | June 1 to July 26, 2022^‡^ | July 1 to August 25, 2022 | August 1 to September 25, 2022 | September 1 to October 26, 2022 | October 1, 2022 to November 25, 2022 |
| --- | --- | --- | --- | --- | --- | --- | --- | --- | --- | --- | --- | --- | --- |
|  | **VE (95% CI) vs unvaccinated** | | | | | | | | | | | |  |
| 65-79-year-olds | N/A | N/A | N/A | N/A | N/A | 85.4% (79.3; 89.8) ^§^ | 76.9% (69.1; 82.8) ^§^ | 66.3% (53.3; 75.7) ^§^ | 65.7% (52.8; 75) ^§^ | 71.6% (57.6; 80.9) ^§¶^ | 74.1% (58.1; 83.9) ^§¶^ | 65.3%  (48.1; 76.8) ^§¶^ | 43.1%  (4.9; 66.0) ^§^ |
| 80+ year-olds | N/A | N/A | N/A | N/A | N/A | 83.5% (73.2; 89.8) | 75.5% (66.1; 82.3) | 68.3% (58.3; 75.9) | 64.9% (42.4; 78.7) | 69.2% (55.6; 78.6) | 77.7% (68.5; 84.3) ^§¶^ | 71.1%  (62.2; 78) ^§¶^ | 64.4% (51.8; 73.7) ^§¶^ |
|  | **rVE (95% CI) vs complete primary vaccination ≥169 days ago** | | | | | | | | | | | |  |
| 65-79-year-olds | N/A | N/A | N/A | N/A | N/A | N/A | 66.0%  (55.2;74.3) ^§^ | 45.8%  (30.3;57.9) ^§^ | 45.7%  (-7.3; 72.5) ^§^ | 47.5%  (-37.9; 80) ^§¶^ | 27.2%  (-215.3;  83.2) ^§¶#^ | -33.0%  (-111.8; 16.5) ^§¶^ | -8.5%  (-69.6; 30.6) ^§¶◊^ |
| 80+ year-olds | N/A | N/A | N/A | N/A | N/A | N/A | 61.3% (48.3; 71.0) | 59.2% (23.6; 78.2) | 53.1% (32.2; 67.5) | 56.5% (32.7; 72.0) | 45.2% (15.5; 64.5) ^¶^ | 37.1%  (15.2; 53.4) ^§¶^ | 34.6% (10.7; 52.1) ^§¶^ |

VE = Vaccine effectiveness; CI = Confidence interval; N/A: Not applicable: In the first six months of the study VE and rVE estimates were not available for death outcome

^†^ Unless otherwise indicated, results are based on pooling VE estimates from Denmark, Luxembourg, Navarre (Spain), Norway and Portugal. (Belgium did not provide estimates of VE against COVID-10 death).

‡ Unless otherwise indicated, results up to June 1 to July 26, 2022 are limited to four sites: Denmark, Navarre (Spain), Norway and Portugal

^§^Navarre (Spain) did not reach 5 events or this dose was still not recommended at this site and did not contribute to the estimate

^¶^ Luxembourg did not reach 5 events or this dose was still not recommended at this site and did not contribute to the estimate

^#^ Denmark did not reach 5 events or this dose was still not recommended at this site and did not contribute to the estimate

^◊^ Norway did not reach 5 events or this dose was still not recommended at this site and did not contribute to the estimate

**Table S5**. Estimated vaccine effectiveness (VE) and relative vaccine effectiveness (rVE) for the second booster dose against COVID-19 death by age group, in overlapping eight-week wide observation intervals from May 2021 (earliest month with available estimates for the 2^nd^ booster) to November 2022, in five^†^ EU/EEA countries. Random effects meta-analysis.

| **Age group** | June 1 to July 26, 2022^‡^ | July 1 to August 25, 2022 | August 1 to September 25, 2022 | September 1 to October 26, 2022 | October 1, 2022 to November 25, 2022 |
| --- | --- | --- | --- | --- | --- |
|  | **VE (95% CI) vs unvaccinated** | | | | |
| 65-79-year-olds | N/A | N/A | ^§¶#◊˜^ | ^§¶#◊˜^ | 77.0%  (47.6; 89.9) ^§¶#◊^ |
| 80+ year-olds | 80.0%  (75; 84) ^§#◊^ | 81.6%  (56.2; 92.3) ^§¶#^ | 85.7%  (44.6; 96.3) ^§¶#^ | 88.6%  (42.1; 97.7) ^§¶#◊^ | 82.9%  (76; 87.9) ^§#◊^ |
|  | **rVE (95% CI) vs complete primary vaccination ≥169 days ago** | | | | |
| 65-79-year-olds | N/A | N/A | ^§¶#◊˜^ | ^§¶#◊˜^ | 61.0%  (29.3; 78.5)  ^§¶◊#^ |
| 80+ year-olds | 68.0%  (60.3; 74.2) ^§¶◊#^ | 58.1%  (44.5; 68.3) ^§¶#^ | 59.9%  (-29.6; 87.6) ^§¶#^ | 75.1%  (-109.4; 97) ^§¶◊#^ | 73.3%  (38.2; 88.4) ^§¶◊^ |
|  | **rVE (95% CI) vs the first booster ≥90 days ago** | | | | |
| 65-79-year-olds | N/A | N/A | ^§¶#◊˜^ | ^§¶#◊˜^ | 74.0%  (60.6; 82.8) ^§¶◊#^ |
| 80+ year-olds | 58.0%  (50.3; 64.5) ^§¶◊#^ | 35.2%  (22.7; 45.7) ^§¶#^ | 37.8%  (-21.3; 68.1) ^§¶#^ | 60.4%  (-90.6; 91.8) ^§¶#^ | 65.0%  (40.9; 79.3) ^§¶◊^ |

VE = Vaccine effectiveness; CI = Confidence interval; N/A: Not applicable before vaccine recommendation was issued.

^†^ Unless otherwise indicated, results are based on pooling VE estimates from Denmark, Luxembourg, Navarre (Spain), Norway and Portugal. Belgium did not provide estimates of VE against COVID-10 death).

^‡^ Results up to June 1 to July 26, 2022 are limited to four sites: Denmark, Navarre (Spain), Norway and Portugal

^§^ Navarre (Spain) did not reach 5 events or this dose was still not recommended at this site and did not contribute to the estimate

^¶^ Luxembourg did not reach 5 events or this dose was still not recommended at this site and did not contribute to the estimate

^#^ Denmark did not reach 5 events or this dose was still not recommended at this site and did not contribute to the estimate

^◊^ Norway did not reach 5 events or this dose was still not recommended at this site and did not contribute to the estimate

^˜^ Portugal did not reach 5 events or this dose was still not recommended at this site and did not contribute to the estimate

## Appendix 8. Measures of heterogeneity from the random-effects meta-analysis pooling site-specific estimates from six EU/EEA countries

**Table S6**. Measures of heterogeneity in the estimation of vaccine effectiveness (VE) for complete primary vaccination (vs. unvaccinated) against COVID-19 hospitalization by age group, in overlapping eight-week wide observation intervals from October 2021 to November 2022 in five^†^ EU/EEA countries (corresponding to estimates in Table 1).

| **Age group** | **VE (95% CI) vs unvaccinated** | | | | | | | | | | | | |
| --- | --- | --- | --- | --- | --- | --- | --- | --- | --- | --- | --- | --- | --- |
|  | October 1 to November 25, 2021^‡^ | November 1 to December 26, 2021^‡^ | December 1, 2021 to January 25, 2022^‡^ | January 1 to February 25, 2022^‡^ | February 1 to March 28, 2022^‡^ | March 1 to April 25, 2022^‡^ | April 1 to May 26, 2022^‡^ | May 1 to June 25, 2022^‡^ | June 1 to July 26, 2022 | July 1 to August 25, 2022 | August 1 to September 25, 2022 | September 1 to October 26, 2022 | October 1, 2022 to November 25, 2022 |
| 65–79 year-olds | I^2^=0% ; tau^2^=0 ; p=0.53 | I^2^=88% ; tau^2^=0.12 ; p<0.01 | I^2^=93% ; tau^2^=0.22 ; p<0.01 | I^2^=94%; tau^2^=0.32; p<0.01 | I^2^=82% ; tau^2^=0.13 ; p<0.01 | I^2^=0% ; tau^2^=0; p=0.67 | I^2^=64% ; tau^2^=0.16 ; p=0.01 | I^2^=83% ; tau^2^=0.57 ; p<0.01 | I^2^=81% ; tau^2^=0.39 ; p<0.01 | I^2^=83% ; tau^2^=0.56 ; p<0.01 | I^2^=0% ; tau^2^=0 ; p=0.64 | I^2^=7% ; tau^2^=0.01 ; p=0.34 | I^2^=0% ; tau^2^=0 ; p=0.36 |
| ≥80-year-olds | I^2^=0% ; tau^2^=0 ; p=0.76 | I^2^= 54%; tau^2^= 0.03; p= 0.09 | I^2^=89%; tau^2^=0.17; p<0.01 | I^2^=56%; tau^2^=0.03; p=0.05 | I^2^=62% ; tau^2^=0.03 ; p=0.09 | I^2^=0% ; tau^2^=0 ; p=0.91 | I^2^=85% ; tau^2^=0.27 ; p<0.01 | I^2^=86% ; tau^2^=0.26 ; p=0.01 | I^2^=34% ; tau^2^=0.30 ; p=0.02 | I^2^=0% ; tau^2^=0 ; p=0.54 | I^2^=0% ; tau^2^=0 ; p=0.67 | I^2^=73% ; tau^2^=0.24 ; p=0.03 | I^2^=73% ; tau^2^=0.26 ; p=0.03 |

VE: Vaccine effectiveness; CI: confidence interval

^†^ Results are based on pooling VE estimates from Denmark, Luxembourg, Navarre (Spain), Norway and Portugal (Belgium did not provide estimates of VE against unvaccinated), unless indicated in equivalent categories in Table 1.

^‡^ Results up to June 1 to July 26, 2022 are limited to four sites: Denmark, Navarre (Spain), Norway and Portugal.

**Table S7**. Measures of heterogeneity in the estimation of vaccine effectiveness and relative vaccine effectiveness (rVE) for the first booster dose against COVID-19 hospitalisation by age group, in overlapping eight-week wide observation intervals from October 2021 to November 2022 in six^†^ EU/EEA countries (corresponding to estimates in Table 2).

| **Age group** | October 1 to November 25, 2021^‡^ | November 1 to December 26, 2021^‡^ | December 1, 2021 to January 25, 2022^‡^ | January 1 to February 25, 2022^‡^ | February 1 to March 28, 2022^‡^ | March 1 to April 25, 2022^‡^ | April 1 to May 26, 2022^‡^ | May 1 to June 25, 2022^‡^ | June 1 to July 26, 2022 | July 1 to August 25, 2022 | August 1 to September 25, 2022 | September 1 to October 26, 2022 | October 1, 2022 to November 25, 2022 |
| --- | --- | --- | --- | --- | --- | --- | --- | --- | --- | --- | --- | --- | --- |
|  | **VE (95% CI) vs unvaccinated** | | | | | | | | | | | |  |
| 65–79 year-olds | I^2^=0% ; tau^2^=0; p=0.47 | I^2^= 74% ; tau^2^=0.14 ; p<0.01 | I^2^=79% ; tau^2^= 0.07; p=0.01 | I^2^=95% tau^2^=0.22; p<0.01 | I^2^=94% ; tau^2^=0.26 ; p<0.01 | I^2^=79% ; tau^2^=0.11 ; p<0.01 | I^2^=12% ; tau^2^=0.01 ; p=0.32 | I^2^=67% ; tau^2^=0.20 ; p=0.01 | I^2^=73% ; tau^2^=0.13 ; p=0.01 | I^2^=56% ; tau^2^=0.08 ; p=0.12 | I^2^=0% ; tau^2^=0 ; p=0.91 | I^2^=35% ; tau^2^=0.06 ; p=0.26 | I^2^=50% ; tau^2^=0.15 ; p=0.15 |
| ≥80-year-olds | I^2^=86% ; tau^2^=0.85 ; p=0.01 | I^2^= 88% ; tau^2^=0.40 ; p<0.01 | I^2^=93% ; tau^2^= 0.24; p<0.01 | I^2^=81% tau^2^= 0.05; p=0.02 | I^2^=55% tau^2^=0.02 ; p=0.07 | I^2^=0% ; tau^2^=0 ; p=0.47 | I^2^=20% ; tau^2^=0.01 ; p=0.30 | I^2^=69% ; tau^2^=0.10 ; p=0.05 | I^2^=77% ; tau^2^=0.11 ; p=0.08 | I^2^=41% ; tau^2^=0.03 ; p=0.23 | I^2^=67% ; tau^2^=0.13 ; p=0.08 | I^2^=72% ; tau^2^=0.17 ; p<0.01 | I^2^=71! ; tau^2^=0.22 ; p<0.01 |
|  | **rVE (95% CI) vs complete primary vaccination ≥169 days ago** | | | | | | | | | | | |  |
| 65–79 year-olds | N/A | N/A | N/A | N/A | N/A | N/A | I^2^=74% ; tau^2^=0.17 ; p=0.01 | I^2^=4% ; tau^2^=0 ; p=0.38 | I^2^=0% ; tau^2^=0 ; p=0.65 | I^2^=55% ; tau^2^=0.06 ; p=0.05 | I^2^=48% ; tau^2^=0.08 ; p=0.13 | I^2^=17% ; tau^2^=0 .02; p=0.27 | I^2^=0% ; tau^2^=0 ; p=0.62 |
| ≥80-year-olds | N/A | N/A | N/A | N/A | N/A | N/A | I^2^=77% ; tau^2^=0.15 ; p=0.07 | I^2^=0% ; tau^2^=0 ; p=0.76 | I^2^=39% ; tau^2^=0.02 ; p=0.26 | I^2^=62% ; tau^2^=0.07 ; p=0.01 | I^2^=0% ; tau^2^=0 ; p=0.76 | I^2^=0% ; tau^2^=0; p=0.92 | I^2^=0% ; tau^2^=0 ; p=0.94 |

VE = Vaccine effectiveness; CI –Confidence interval; N/A: Not applicable: In the first six months of the study

^†^Results are based on pooling VE estimates from Belgium, Denmark, Luxembourg, Navarre (Spain), Norway and Portugal, except absolute VE estimates (using the unvaccinated as a reference) which do not include Belgium, unless indicated in equivalent categories in Table 2.

^‡^ Results up to June 1 to July 26, 2022 are limited to four sites: Denmark, Navarre (Spain), Norway and Portugal

**Table S8**. Measures of heterogeneity in the estimation of vaccine effectiveness (VE) and relative vaccine effectiveness (rVE) for the second booster dose against COVID-19 hospitalization by age group, in overlapping eight -week wide observation intervals from May 2021 (earliest month with available estimates for the 2nd booster) to November 2022, in six^†^ EU/EEA countries (corresponding to estimates in Table 3).

| **Age group** | June 1 to July 26, 2022^‡^ | July 1 to August 25, 2022 | August 1 to September 25, 2022 | September 1 to October 26, 2022 | October 1, 2022 to November 25, 2022 |
| --- | --- | --- | --- | --- | --- |
|  | **VE (95% CI) vs unvaccinated** | | | | |
| 65–79 year-olds | N/A | N/A | I^2^=0% ; tau^2^=0 ; p=1.00 | I^2^=16% ; tau^2^=0.04 ; p=0.28 | I^2^=0% ; tau^2^=0 ; p=0.42 |
| ≥80-year-olds | I^2^=0% ; tau^2^=0 ; p=1.00 | I^2^=0% ; tau^2^=0 ; p=.041 | I^2^=51% ; tau^2^=0.05 ; p=0.15 | I^2^=70% ; tau^2^=0.19 ; p=0.01 | I^2^=0% ; tau^2^=0 ; p=0.53 |
|  | **rVE (95% CI) vs complete primary vaccination ≥169 days ago** | | | | |
| 65–79 year-olds | N/A | N/A | I^2^=0% ; tau^2^=0 ; p=1.00 | I^2^=0% ; tau^2^=0 ; p=0.59 | I^2^=0% ; tau^2^=0 ; p=0.62 |
| ≥80-year-olds | I^2^=0% ; tau^2^=0 ; p=1.00 | I^2^=0% ; tau^2^=0 ; p=0.79 | I^2^=26% ; tau^2^=0.02 ; p=0.25 | I^2^=0% ; tau^2^=0; p=0.78 | I^2^=47% ; tau^2^=0.08 ; p=0.09 |
|  | **rVE (95% CI) vs the first booster≥90 days ago** | | | | |
| 65–79 year-olds | N/A | N/A | I^2^=0% ; tau^2^=0 ; p=1.00 | I^2^=0% ; tau^2^=0 ; p=0.86 | I^2^=48% ; tau^2^=0.09 ; p=0.24 |
| ≥80-year-olds | I^2^=0% ; tau^2^=0 ; p=1.00 | I^2^=91% ; tau^2^=0.12; p<0.01 | I^2^=90% ; tau^2^=0.14 ; p<0.01 | I^2^=54% ; tau^2^=0.03 ; p=0.12 | I^2^=90% ; tau^2^=0.28 ; p<0.01 |

VE = Vaccine effectiveness; CI –Confidence interval. N/A: Not applicable: before vaccine recommendation was issued

^†^ Results are based on pooling VE estimates from Belgium, Denmark, Luxembourg, Navarre (Spain), Norway and Portugal, except absolute VE estimates (using the unvaccinated as a reference) which do not include Belgium, unless indicated in equivalent categories in Table 3.

^‡^ Results up to June 1 to July 26, 2022 are limited to four sites: Denmark, Navarre (Spain), Norway and Portugal

**Table S9**. Measures of heterogeneity in the estimation of vaccine effectiveness (VE) for primary vaccination (vs. unvaccinated) against COVID-19 death by age group, in overlapping eight-week wide observation intervals from March 2022 to November 2022, in five^†^ EU/EEA countries (corresponding to estimates in Table S1).

| **Age group** | **VE (95% CI) vs.unvaccinated** | | | | | | | | | | | | |
| --- | --- | --- | --- | --- | --- | --- | --- | --- | --- | --- | --- | --- | --- |
|  | October 1 to November 25, 2021^‡^ | November 1 to December 26, 2021^‡^ | December 1, 2021 to January 25, 2022^‡^ | January 1 to February 25, 2022^‡^ | February 1 to March 28, 2022^‡^ | March 1 to April 25, 2022^‡^ | April 1 to May 26, 2022^‡^ | May 1 to June 25, 2022^‡^ | June 1 to July 26, 2022 | July 1 to August 25, 2022 | August 1 to September 25, 2022 | September 1 to October 26, 2022 | October 1, 2022 to November 25, 2022 |
| 65-79-year-olds | N/A | N/A | N/A | N/A | N/A | I^2^=44% ; tau^2^=0.06 ; p=0.20 | I^2^=58% ; tau^2^=0.19 ; p=0.11 | I^2^=0% ; tau^2^=0 ; p=0.93 | I^2^=0% ; tau^2^=0 ; p=0.37 | I^2^=52% ; tau^2^=0.22 ; p=0.14 | I^2^=80% ; tau^2^=1.02; p=0.01 | I^2^=0% ; tau^2^=0 ; p=1.00 | I^2^=0% ; tau^2^=0 ; p=1.00 |
| 80+ year-olds | N/A | N/A | N/A | N/A | N/A | I^2^=22% ; tau^2^=0.01 ; p=0.28 | I^2^=85% ; tau^2^=0.36 ; p<0.01 | I^2^=93% ; tau^2^=1.52 ; p=0.02 | I^2^=66% ; tau^2^=0.17 ; p=0.13 | I^2^=87% ; tau^2^=0.52 ; p=0.01 | I^2^=0% ; tau^2^=0 ; p=0.63 | I^2^=43% ; tau^2^=0.09 ; p=0.15 | I^2^=72% ; tau^2^=0.28 ; p=0.06 |

VE = Vaccine effectiveness; CI = confidence interval; N/A: Not applicable: In the first six months of the study VE estimates were not available for death outcome

^†^ Results are based on pooling VE estimates from Denmark, Luxembourg, Navarre (Spain), Norway and Portugal. (Belgium did not provide estimates of VE against COVID-10 death), unless indicated in equivalent categories in Table S1.

^‡^ Results up to June 1 to July 26, 2022 are limited to four sites: Denmark, Navarre (Spain), Norway and Portugal

**Table S10**. Measures of heterogeneity in the estimation of vaccine effectiveness (VE) and relative vaccine effectiveness (rVE) for the first booster dose against COVID-19 death by age group, in overlapping eight-week wide observation intervals from October 2021 to November 2022, in five^†^ EU/EEA countries (corresponding to estimates in Table S2).

| **Age group** | October 1 to November 25, 2021^‡^ | November 1 to December 26, 2021^‡^ | December 1, 2021 to January 25, 2022^‡^ | January 1 to February 25, 2022^‡^ | February 1 to March 28, 2022^‡^ | March 1 to April 25, 2022^‡^ | April 1 to May 26, 2022^‡^ | May 1 to June 25, 2022^‡^ | June 1 to July 26, 2022 | July 1 to August 25, 2022 | August 1 to September 25, 2022 | September 1 to October 26, 2022 | October 1, 2022 to November 25, 2022 |
| --- | --- | --- | --- | --- | --- | --- | --- | --- | --- | --- | --- | --- | --- |
|  | **VE (95% CI) vs unvaccinated** | | | | | | | | | | | |  |
| 65-79-year-olds | N/A | N/A | N/A | N/A | N/A | I^2^=50% ; tau^2^=0.05 ; p=0.15 | I^2^=0% ; tau^2^=0 ; p=0.57 | I^2^=3% ; tau^2^=0.01 ; p=0.35 | I^2^=0% ; tau^2^=0 ; p=0.57 | I^2^=0% ; tau^2^=0 ; p=0.53 | I^2^=23% ; tau^2^=0.04 ; p=0.26 | I^2^=0% ; tau^2^=0 ; p=0.57 | I^2^=0% ; tau^2^=0 ; p=0.95 |
| 80+ year-olds | N/A | N/A | N/A | N/A | N/A | I^2^=86% ; tau^2^=0.19 ; p<0.01. | I^2^=49% ; tau^2^=0.05 ; p=0.11 | I^2^=19% ; tau^2^=0.02 ; p=0.29 | I^2^=77% ; tau^2^=0.17 ; p<0.01 | I^2^=45% ; tau^2^=0.07 ; p=0.04 | I^2^=38% ; tau^2^=0.04 ; p=0.17 | I^2^=0% ; tau^2^=0 ; p=0.50 | I^2^=6% ; tau^2^=0.01 ; p=0.34 |
|  | **rVE (95% CI) vs complete primary vaccination ≥169 days ago** | | | | | | | | | | | |  |
| 65-79-year-olds | N/A | N/A | N/A | N/A | N/A | N/A | I^2^=0% ; tau^2^=0 ; p=0.55 | I^2^=0% ; tau^2^=0 ; p=0.40 | I^2^=65% ; tau^2^=0.24 ; p=0.05 | I^2^=86% ; tau^2^=0.62 ; p<0.01 | I^2^=91% ; tau^2^=1.51 ; p<0.02 | I^2^=0% ; tau^2^=0; p=0.41 | I^2^=0% ; tau^2^=0 ; p=0.59 |
| 80+ year-olds | N/A | N/A | N/A | N/A | N/A | N/A | I^2^=29% ; tau^2^=0.03 ; p=0.18 | I^2^=74% ; tau^2^=0.27 ; p=0.02 | I^2^=58% ; tau^2^=0.08 ; p=0.02 | I^2^=62% ; tau^2^=0.13 ; p=0.01 | I^2^=37% ; tau^2^=0.07 ; p=0.19 | I^2^=0% ; tau^2^=0; p=0.43 | I^2^=0% ; tau^2^=0 ; p=0.81 |

VE = Vaccine effectiveness; CI = Confidence interval; N/A: Not applicable: In the first six months of the study VE and rVE estimates were not available for death outcome

^†^Results are based on pooling VE estimates from Denmark, Luxembourg, Navarre (Spain), Norway and Portugal (Belgium did not provide estimates of VE against COVID-10 death), unless indicated in equivalent categories in Table S2.

^‡^ Results up to June 1 to July 26, 2022 are limited to four sites: Denmark, Navarre (Spain), Norway and Portugal

**Table S11**. Measures of heterogeneity in the estimation of vaccine effectiveness (VE) and relative vaccine effectiveness (rVE) for the second booster dose against COVID-19 death by age group, in overlapping eight-week wide observation intervals from May 2021 (earliest month with available estimates for the 2^nd^ booster) to November 2022, in five^†^ EU/EEA countries (corresponding to estimates in Table S3).

| **Age group** | June 1 to July 26, 2022^‡^ | July 1 to August 25, 2022 | August 1 to September 25, 2022 | September 1 to October 26, 2022 | October 1, 2022 to November 25, 2022 |
| --- | --- | --- | --- | --- | --- |
|  | **VE (95% CI) vs unvaccinated** | | | | |
| 65-79-year-olds | N/A | N/A | § | § | I^2^=0% ; tau^2^=0 ; p=1.00 |
| 80+ year-olds | I^2^=0% ; tau^2^=0 ; p=1.00 | I^2^=85% ; tau^2^=0.34 ; p=0.01 | I^2^=93% ; tau^2^=0.89 ; p<0.01 | I^2^=92% ; tau^2^=1.27 ; p<0.01 | I^2^=0% ; tau^2^=0 ; p=0.92 |
|  | **rVE (95% CI) vs complete primary vaccination ≥169 days ago** | | | | |
| 65-79-year-olds | N/A | N/A | § | § | I^2^=0% ; tau^2^=0 ; p=1.00 |
| 80+ year-olds | I^2^=0% ; tau^2^=0 ; p=1.00 | I^2^=0% ; tau^2^=0 ; p=0.34 | I^2^=88% ; tau^2^=0.63; p<0.01 | I^2^=95% ; tau^2^=2.24 ; p<0.01 | I^2^=72% ; tau^2^=0.27 ; p=0.06 |
|  | **rVE (95% CI) vs the first booster ≥90 days ago** | | | | |
| 65-79-year-olds | N/A | N/A | § | § | I^2^=0% ; tau^2^=0 ; p=1.00 |
| 80+ year-olds | I^2^=0% ; tau^2^=0 ; p=1.00 | I^2^=0% ; tau^2^=0 ; p=0.93 | I^2^=82% ; tau^2^=0.19 ; p=0.02 | I^2^=94% ; tau^2^=1.21 ; p<0.01 | I^2^=75% ; tau^2^=0.11 ; p=0.05 |

VE = Vaccine effectiveness; CI = Confidence interval; N/A: Not applicable before vaccine recommendation was issued.

^†^ Results are based on pooling VE estimates from Denmark, Luxembourg, Navarre (Spain), Norway and Portugal (Belgium did not provide estimates of VE against COVID-10 death), unless indicated in equivalent categories in Table S3.

^‡^ Results up to June 1 to July 26, 2022 are limited to four sites: Denmark, Navarre (Spain), Norway and Portugal.

§ Not Possible to estimate due to low number of events (less than 15 events)
